# Molecular Engineering of Terminus, Conjugation, and Energetics for Thermally Stable Inverted Perovskite Solar Cells

**DOI:** 10.1021/jacs.5c09669

**Published:** 2025-08-22

**Authors:** Jiaonan Sun, Jiarong Wang, Ze-Fan Yao, Leyu Bi, Xiaofei Ji, Jia Wang, Xiaofeng Huang, Ming Liu, Kaikai Liu, Francis R. Lin, Bin Kan, Qiang Fu, Alex K.-Y. Jen

**Affiliations:** † Department of Materials Science and Engineering, 53025 City University of Hong Kong, Kowloon, Hong Kong 999077, China; ‡ Department of Chemistry, 53025 City University of Hong Kong, Kowloon, Hong Kong 999077, China; § School of Chemical Engineering and Light Industry, 47870 Guangdong University of Technology, Guangzhou 510006, China; ∥ Hong Kong Institute for Clean Energy, 53025 City University of Hong Kong, Kowloon, Hong Kong 999077, China; ⊥ College of Chemistry and Molecular Engineering, 12465 Peking University, Beijing 100871, China; ▽ The Interdisciplinary Research Center, Shanghai Advanced Research Institute, Chinese Academy of Sciences, Shanghai, Pudong 201210, China; g School of Materials Science and Engineering, National Institute for Advanced Materials, 12538 Nankai University, Tianjin 300350, China; h State Key Laboratory of Marine Pollution, 53025 City University of Hong Kong, Kowloon, Hong Kong 999077, China

## Abstract

Low-dimensional (LD)/three-dimensional (3D) heterostructure perovskite solar cells (PSCs) have achieved a power conversion efficiency (PCE) greater than 26%. However, the use of some ionic interfacial passivation materials in the construction of LD perovskites compromises device stability, as they can induce ion diffusion, particularly under high temperatures and light stress. In this study, we substitute the ammonium terminus (R-NH_3_^+^) of conventional passivators with a carbamate terminus (R-NH-(CO)­OR) and synthesized carbamate molecules featuring phenyl (PEA-Boc) and naphthalimide (ND-Boc) scaffolds. Through modulating the ionic terminus and enlarging the conjugated backbone of the passivation materials, the interlayer diffusion across PSCs was effectively inhibited. Moreover, the ND-Boc with electron-accepting moieties optimizes the band energy alignment, reduces defect density, and facilitates interfacial electron transfer of PSCs. As a result, the small-area target PSCs (0.04 cm^2^) and mini-modules (aperture area of 15.45 cm^2^) achieved a PCE of 26.04% and 21.83%, respectively. Notably, the encapsulated ND-Boc-based PSC maintained 96.7% of its initial PCE after being tracked at a maximum power point for 1500 h at 85 °C under argon (ISOS-L-2I protocol). Our strategy offers a simple and generally applicable passivation method for fabricating efficient and robust PSCs to facilitate their practical applications.

## Graphical abstract

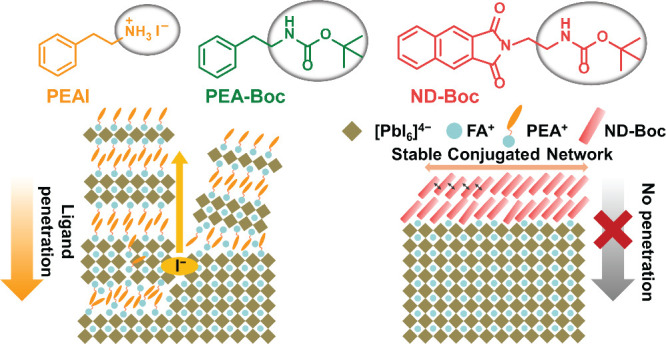

## Introduction

With the rapid advancement of perovskite solar cells (PSCs), their power conversion efficiencies (PCEs) have reached beyond 26%, comparable to commercial silicon solar cells.^1–7^ However, their operational stability under high temperatures and light has become increasingly scrutinized, emerging as a key factor that restricts their further development for commercialization.^8–12^ For high-performance PSCs, defect passivation is one of the most effective strategies to improve device efficiency.^6,13–15^ Many current passivation materials, e.g., metal cations,^16^ Lewis acid and base,^17,18^ low-dimensional (LD) perovskite-forming ligands,^19–22^ and polymers,^23^ have significantly enhanced the PCE of PSCs.^24–27^

In inverted PSCs, ionic passivation materials such as piperazinium iodide (PI), phenethylammonium iodide (PEAI, Figure 1a) and their derivatives are commonly used to improve efficiency, which is capable of forming LD/three-dimensional (3D) heterostructures at the upper interface of perovskite or passivating the surface via ionic interactions.^28–32^ However, under light and heat exposure, small-sized passivators can move within or across the “soft” perovskite lattice, which tends to deteriorate PSC performance during operation. For example, perovskite films utilizing PEAI have photoluminescence (PL) intensity decay and shortened PL lifetime under heating or applied bias.^30,33^ The PEA^+^ cation can also generate neutral amines (basic) to attack the FAI component in CsFA-based perovskite.^34–36^ With increased heat exposure, the degradation of PEAI-based devices could be even faster than that of control devices.^37,38^ Several strategies have been reported addressing these issues. For example, removing the van der Waals “gap” to build DJ-phase 2D perovskites with double cation binding sites enhanced the thermal stability compared to the RP-phase counterparts.^39^ Perovskitoids with edge- and face-sharing networks, exhibiting an organic–inorganic framework distinct from conventional 2D perovskites with corner-shared lattices, were recently developed to impede cation migration.^40^ On the other hand, non-2D perovskite ligands, such as the ortho-isomer of phenylenediammonium, prevent the formation of 2D perovskite due to the steric effect, representing a new approach to improve the device stability while maintaining the passivation effect.^41,42^

**Figure 1:**
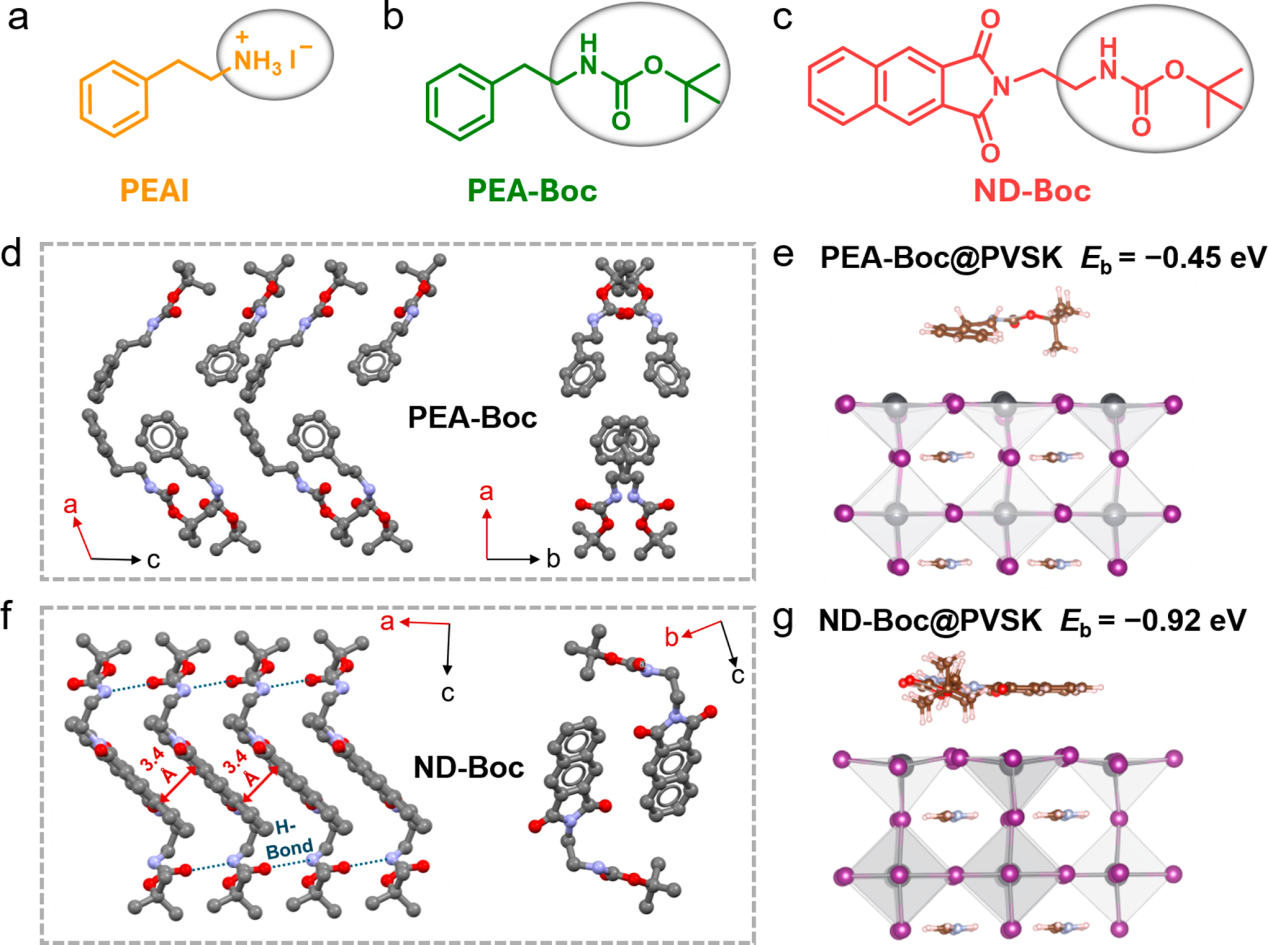
Molecular structures of (a) PEAI, (b) PEA-Boc, and (c) ND-Boc. (d) Single-crystal structure of PEA-Boc. (e) Optimized PEA-Boc@PbI_2_-rich (100) perovskite interface. (f) Single-crystal structure of ND-Boc. (g) Optimized ND-Boc@ PbI_2_-rich (100) perovskite interface. The DFT calculation is based on GGA/PBE.

Considering the synthetically challenging steric design of non-2D ligands, we are motivated by the exploration of simple and straightforward passivation strategies that can further improve the efficiency and stability of PSCs. Therefore, we targeted the ammonium terminus of commonly used small molecules such as PEAI and developed a universal Boc substitution method to “block” the ionic terminus of passivators. Initially, we synthesized a phenethylcarbamate (PEA-Boc, Figure 1b) molecule. PEA-Boc interfacial layer improved device thermal stability but resulted in a reduced passivation effect compared to PEAI. To further optimize the interfacial passivation and energetics, we introduced naphthalimide group and synthesized *tert*-butyl (2-(1,3-dioxo-1,3-dihydro-2H-benzo­[f]­isoindol-2-yl)­ethyl)­carbamate (ND-Boc, Figure 1c). ND-Boc integrates the advantages of a Boc-substituted terminus, *n*-character from electron-accepting moieties, and an extended conjugated backbone, restricting the interlayer diffusion of passivators and anions, as evidenced by time-of-flight secondary ion mass spectrometry (ToF-SIMS) and atomic force microscope-infrared spectroscopy (AFM-IR). As a result, ND-Boc-based PSCs achieved a champion efficiency of 26.04% and mini-modules (5 × 5 cm^2^) reached an aperture-area PCE of 21.83% with an aperture area of 15.45 cm^2^. ND-Boc-passivated PSCs exhibited excellent long-term stability under 65 and 85 °C heating, with a negligible decrease of their PCEs after 3768 and 2160 h, respectively. Notably, the ND-Boc-passivated PSC maintained 96.7% of its initial efficiency after 1500 h of maximum power point tracking (MPPT) at 85 °C.

## Results and Discussion

As shown in Figure S1, the synthesis of PEA-Boc involved a two-step reaction starting from PEAI, while ND-Boc was synthesized through a one-step conversion from naphthalenedicarboxylic anhydride. Single crystals of these molecules were obtained by the solvent evaporation method (Figure 1d and 1f, Tables S1, S2). The single-crystal structure of PEA-Boc showed a herringbone packing arrangement, with the benzene rings preferentially aligned in a perpendicular direction. Note that the single-crystal structure of PEA-Boc has been reported previously.^43^ On the other hand, the single crystal of ND-Boc displayed strong planar π-π stacking with the neighboring planes separated by 3.4 Å, but with multiple H-bonds at a distance of 3.0 Å. Notably, the conjugated planes of the naphthalimide units only partially overlap, indicating the formation of *J*-aggregates. In addition, we employed density functional theory (DFT) calculations to examine how the passivators interacted with the perovskite. When these passivators exhibit face-on configuration on the perovskite surface, the binding energy (*E*_b_) of these with the PbI_2_-rich (100) phase of perovskites can be calculated (Figure 1e and 1g). PEA-Boc molecule shows an *E*_b_ of −0.45 eV, while ND-Boc with multiple functional groups shows a larger *E*_b_ of −0.92 eV. In edge-on configuration, ND-Boc also had larger *E*_b_ and directional interaction from the C=O of the naphthalimide moiety (Figure S2). Both single crystal analysis and the DFT calculation of binding energy illustrate that our molecular design with the extended conjugation and naphthalimide moieties contributes to better molecular packing and stronger interactions with the upper surface of perovskites. In terms of the intrinsic material properties, the results from thermogravimetric analysis (TGA, Figure S3) showed the decomposition temperatures of PEA-Boc and ND-Boc are 155 and 211 °C, respectively, much higher than the aging temperatures of PSCs.

To further study the interaction between perovskite and passivators, we mixed these materials with PbI_2_ in a molar ratio of 1:1 and spin-coated the mixture into films to compare with those made from pristine PbI_2_ and a mixture of PEAI+PbI_2_. UV–vis spectroscopy revealed that the PbI_2_ thin films exhibited a distinct absorption edge at 501 nm (Figure 2a). Following the introduction of PEA-Boc, the absorbance decreased, indicating a mild interaction between PEA-Boc and PbI_2_ that resulted in suppressed crystalline PbI_2_.^44^ The mixture of ND-Boc and PbI_2_ exhibited a slight shift in the absorption peak from 501 to 495 nm, demonstrating stronger interactions between PbI_2_ and ND-Boc. This new peak at 415 nm could be assigned to the absorption characteristics of ND-Boc itself (Figure S4). When PEAI was mixed with PbI_2_, the resulting film exhibited excitonic peaks at 517 nm, which can be attributed to the formation of 2D (PEA)_2_PbI_4_ perovskite. X-ray powder diffraction (XRD) analysis of these samples revealed a consistent trend (Figure 2b). The PbI_2_ peak at 2θ = 12.9° has only decreased in intensity after the introduction of PEA-Boc. In the mixed ND-Boc and PbI_2_ thin films, the characteristic PbI_2_ peak at 12.97° shifted slightly to 12.77°. Two small new peaks at 10.3° and 7.7° indicate a different packing arrangement of ND-Boc interacting with PbI_2_. ND-Boc did not display any diffraction peaks due to its intrinsic amorphous nature (Figure S5). We attempted to grow the single crystal by mixing ND-Boc with PbI_2_. However, separate crystals of ND-Boc and PbI_2_ were obtained rather than a combined single crystal. As a neutral species, ND-Boc cannot form ionic bonds with the PbI_2_ lattice. Therefore, we infer that ND-Boc mostly remains in its molecular form on top of perovskites. Film with PEAI+PbI_2_ mixture formed (PEA)_2_PbI_4_ 2D perovskites, with residual PbI_2_ present. The interactions of ND-Boc with the perovskite components, specifically PbI_2_ and FAI, can be further validated through nuclear magnetic resonance (NMR) analysis (Figure 2c). After mixing ND-Boc with PbI_2_, the aryl-H close to the C=O upfield shifted slightly from 8.509 to 8.504 ppm. After mixing ND-Boc with FAI, the NH peaks at 8.824 ppm from FAI shifted to 8.834 ppm, and the central C–H of FAI also downfield shifted from 7.841 to 7.853 ppm. Additionally, ND-Boc also exhibits changes in N–H and C=O stretching, characterized by peak broadening during its interaction with FAI or PbI_2_, as evidenced by attenuated total reflectance-Fourier transform infrared (ATR-FTIR) spectroscopy (Figure S6–S8).

**Figure 2:**
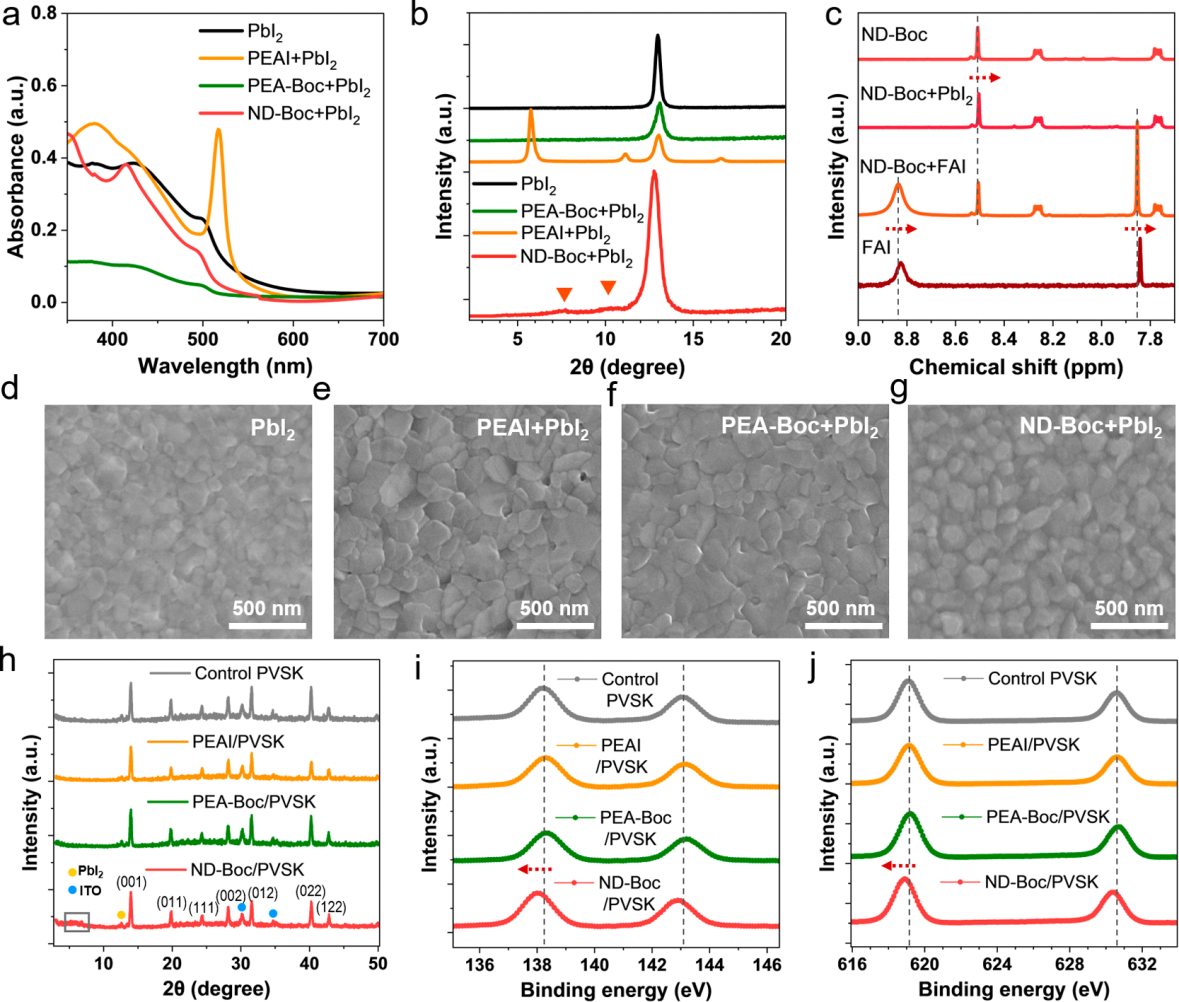
(a) UV–vis spectra of thin films composed of PbI_2_, PEAI mixed with PbI_2_, PEA-Boc mixed with PbI_2_, and ND-Boc mixed with PbI_2_ in 1:1 molar ratio. (b) X-ray diffraction patterns of thin films composed of PbI_2_, PEAI mixed with PbI_2_, PEA-Boc mixed with PbI_2_, and ND-Boc mixed with PbI_2_ in 1:1 molar ratio (c) ^1^H NMR of ND-Boc, ND-Boc mixed with PbI_2_, and ND-Boc mixed with FAI solution. The solvent for NMR is DMSO-*d*_6_. (d–g) SEM of (d) pristine, (e) PEAI-treated, (f) PEA-Boc-treated, and (g) ND-Boc-treated PbI_2_ film. (h) XRD pattern of the control and PEAI/PEA-Boc/ND-Boc-treated perovskite films. (i, j) XPS spectra of the (i) Pb 4f core level and (j) I 3d core level of the control and PEAI/PEA-Boc/ND-Boc-treated perovskite films.

To further correlate these interactions with perovskite surface morphology, we conducted scanning electron microscopy (SEM) to examine PbI_2_ films and PbI_2_ films treated with PEAI, PEA-Boc, and ND-Boc solutions (Figure 2d-2g). PEAI converted the original rough morphology of PbI_2_ into a flake-like structure characterized by crystalline facets indicative of 2D perovskite structures. PEA-Boc altered the rigid inorganic structures into amorphous planar organic structures. Notably, due to stronger interactions with PbI_2_, ND-Boc disrupted the interface between PbI_2_ to produce island-like rounded polyhedrons.

Building on the understanding of chemical interactions among PbI_2_, FAI, and passivators, we prepared perovskite thin films with a composition of Cs_0.05_FA_0.95_PbI_3_ (the same as that used in PSCs) and investigated the surface properties of perovskite films after 1 mg mL^–1^ of PEAI, PEA-Boc and ND-Boc treatments. First, XRD analysis identified a new broad peak in the 4°-7° range, which is due to interactions between ND-Boc and perovskite (mostly FAI and PbI_2_) since ND-Boc does not exhibit any peaks in the XRD pattern (Figure 2h and S9). Additionally, PEAI-based 2D perovskite was observed on the surface of the perovskite film when the concentration of PEAI was increased to 5 mg mL^–1^ (Figure S10). The XRD patterns of the bulk perovskite material in the region of 2θ > 10° exhibit consistent facets and intensity for all surface treatments. Subsequently, surface-sensitive X-ray photoelectron spectroscopy (XPS) was employed to analyze the binding energy regions of N 1s and O 1s, confirming the presence of nitrogen and oxygen elements from the (CO)-NR-(CO) and RNH-(CO) functional groups in ND-Boc (Figure S11). More importantly, Pb 4f spectra of XPS revealed a reduced binding energy of 0.2 eV for ND-Boc-treated perovskite films, indicating that the lone-pair electrons on C=O in ND-Boc interact with undercoordinated Pb^2+^, resulting in an increased electron density on Pb (Figure 2i and 2j). Concurrently, the strengthened interaction between C=O and Pb leads to a higher electron density on I^–^, causing the I 3d_5/2_ peaks of ND-Boc passivated perovskite film to shift from 619.1 to 618.9 eV. The shifts of Pb 4f and I 3f XPS spectra further corroborate the interaction between ND-Boc and the perovskite surface.^45^ The introduction of an additional ND-Boc passivation layer also increased the contact angle from 47° to 79°, indicating a more hydrophobic surface facilitated by ND-Boc (Figure S12). Regarding the morphology of the perovskite upper surface, AFM and SEM showed similar surface roughness on the surface of ND-Boc modified perovskites compared with control films (Figure S13–14).

We further employed *in situ* PL measurement to probe the passivation mechanisms associated with various passivators (Figure 3a-3e). During the annealing process of perovskite films right after the spin-coating of passivator solutions, the PL was monitored over the first 10 s. From the PL heat map, we observe an initial increase of PL intensity originating from surface defect passivation and the following decay of intensity due to thermal quenching (thermally activated carrier trapping and nonradiative decay via lattice vibration at elevated temperature) at the 100 °C annealing temperature.^46,47^ PEAI-treated perovskite film reveals the highest PL intensity of 2.8 × 10^4^ due to the generation of 2D perovskite atop the 3D perovskite surface. Without 2D perovskite formation, the ND-Boc-treated perovskite film shows the second-highest PL intensity, primarily attributed to ND-Boc interactions with PbI_2_ and FAI, reducing surface defects. In contrast, the PEA-Boc-treated perovskite film did not show any PL enhancement compared to control films, which is attributed to the lack of an ammonium terminus necessary for 2D perovskite formation or effective functional groups for surface interactions. The steady-state PL was also measured for the final film after 10 min of thermal annealing (Figure S15). ND-Boc reveals the highest PL intensity, increasing from 5.4 × 10^4^ to 8.4 × 10^4^.

**Figure 3:**
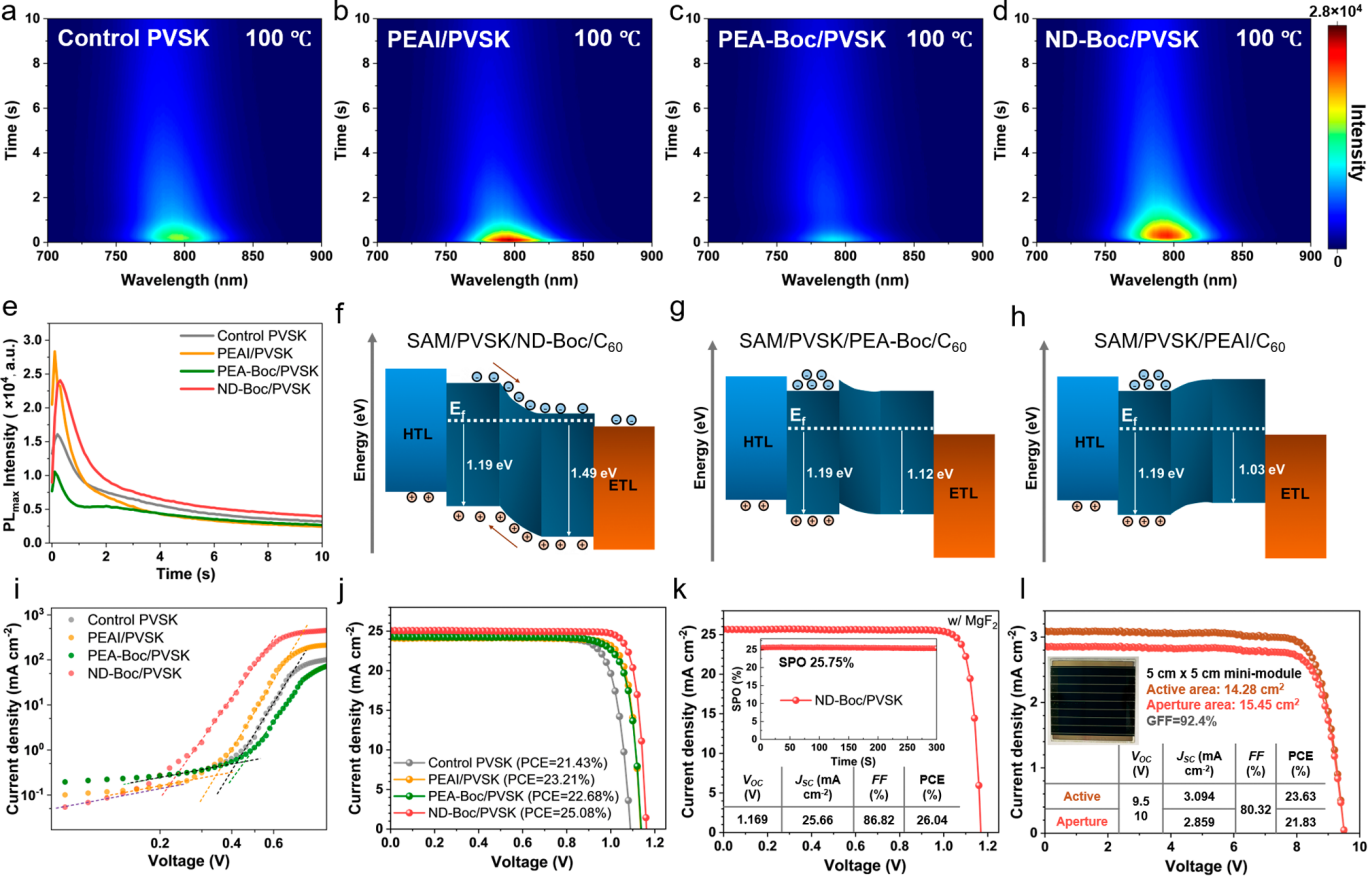
(a–d) Heat maps of *in situ* PL for (a) control, (b) PEAI-treated, (c) PEA-Boc-treated, and (d) ND-Boc-treated perovskite films during the annealing process at 100 °C. (e) Peak intensity evolution of the maximum peak extracted from *in* *situ* PL spectra. (f–h) Schematic illustration of interfacial band bending for PEAI-/PEA-Boc-/ND-Boc-treated perovskite films based on data extracted from UPS spectra. (i) SCLC of electron-only devices. (j) Representative *J–V* curves of the control PSC and PSCs treated by PEAI/PEA-Boc/ND-Boc. (k) The champion *J–V* curve of the PSC treated by ND-Boc. The inset is the SPO. (l) *J–V* curve of a perovskite mini-module based on ND-Boc post-treatment with an active area of 14.28 cm^2^ or an aperture area of 15.45 cm^2^. The inset shows a photograph of the 5 cm × 5 cm mini-module.

Concerning interfacial charge transfer and energy alignment, ultraviolet photoelectron spectroscopy (UPS) was utilized to determine the work function of the top surface of perovskite films after passivation (Figures 3f-h and S16). The work function increased from −4.22 eV to −3.75 eV due to the n-doping effect induced by ND-Boc, confirming the electron-accepting properties of the ND-Boc interfacial layers. This modification facilitates electron transfer with interfacial band bending and reduces charge recombination. Additionally, Kelvin probe force microscopy (KPFM), shown in Figure S17, also revealed an increased contact potential difference (shallower energy levels) for ND-Boc passivated perovskite films, aligning closely with the work function extracted from UPS. Besides, the space-charge-limited current (SCLC) analysis of electron-only devices demonstrates that the ND-Boc-treated perovskite film exhibits the lowest trap-filling limited voltage (*V*_TFL_) of 0.21 V, representing the lowest trap density of 1.37 × 10^15^ cm^–3^ among all the passivators (Figure 3i and Table S3).

After obtaining an in-depth understanding of the effects of these passivators on perovskite surfaces, we fabricate positive-intrinsic-negative (*p-i-n*) inverted PSCs with different surface passivation. The device configuration is ITO/SAM/Cs_0.05_FA_0.95_PbI_3_/C_60_/bathocuproine (BCP)/Ag (Figure S18). As shown in Figure 3j, the representative control PSC revealed a PCE of 21.43%. PEA-Boc- and PEAI-based PSCs exhibited moderate improvements of PCE to 22.68% and 23.21%, respectively, primarily due to the improved open-circuit voltage (*V*_*OC*_) and fill-factor (*FF*) (Statistics in Figure S19). Among all the surface treatments, ND-Boc reveals the largest enhancement in PSC efficiency. The best-performing ND-Boc-based PSC with MgF_2_ antireflection coating showed a PCE of 26.04% with a *V*_*OC*_ of 1.169 V, short-circuit current density (*J*_*SC*_) of 25.66 mA cm^–2^, and *FF* of 86.82% (Figure 3k). The stabilized power output (SPO) of the champion PSCs is 25.75%, under a bias of *V*_max_ at 1.06 V, as shown in the inset of Figure 3k. The integrated *J*_*SC*_ (25.06 mA cm^–2^) from the external quantum efficiency (EQE) spectrum of champion PSCs also matches well with that of *J–V* scans (Figure S20). Compared to the unannealed sample, the fill-factor of the ND-Boc-based devices shows a significant improvement following annealing (Figure S21). This enhancement may result from heat-induced optimization of the π-π stacking, which subsequently increases interlayer conductivity. To demonstrate its scalability to larger-sized devices for practical applications, we also fabricated a perovskite solar mini-module (5 × 5 cm^2^) with an active area of 14.28 cm^2^ or an aperture area of 15.45 cm^2^. This mini-module demonstrated an active area efficiency of 23.63% and an aperture-area efficiency of 21.83% (Figure 3l). For wide-bandgap solar cells, our initial fabrication of 1.8 eV perovskite devices demonstrates that ND-Boc passivation enhances the PCE from 15.76% to 18.68% (Figure S22 and Table S4), confirming its effectiveness as a universal approach for improving PSC performance.

A series of aging tests were conducted to systematically investigate the relationship between our molecular engineering approach and overall device stability to address critical ion diffusion and migration, charge extraction efficiency, and interfacial morphological degradation. The unencapsulated ND-Boc and PEA-Boc passivated PSCs with a BCP buffer layer maintained their initial efficiencies nearly unchanged after continuous heating at 65 °C for 3768 h (ISOS-D-2I protocol), demonstrating the best thermal stability (Figure 4a). In contrast, the PEAI-modified and control PSCs only retained 90.8% and 95.7% of their initial efficiencies, respectively. Control PSCs experienced a more severe burn-in effect within the first 300 h. However, their final PCE is better than that of PEAI-based PSCs. Although PEAI-passivated and control PSCs demonstrate lower performance than PSCs with target surface treatments, only minor differences were observed during the relatively mild 65 °C thermal testing, which included an aging time of up to five months. Therefore, we increase the temperature of thermal heating from 65 to 85 °C to accelerate the aging process (Figure 4b). Under 85 °C thermal heating, the bathocuproine (BCP) buffer layer was replaced with a tin oxide layer fabricated using atomic-layer deposition. The ND-Boc-based PSCs revealed no visible degradation and retained close to 100% of their initial PCE after 2160 h heating at 85 °C (corresponding to the ISOS-D-2I level accelerated aging). PEA-Boc-based PSC also showed relatively stable performance, retaining 85.0% of its initial PCE after heating for 2160 h. In contrast, control PSCs revealed significant decay, reaching T_80_ after 1080 h. Moreover, the PEAI passivated PSCs decay even faster than the control PSCs, with performance declining to 4.1% of that of the pristine PSCs after 960 h.

**Figure 4:**
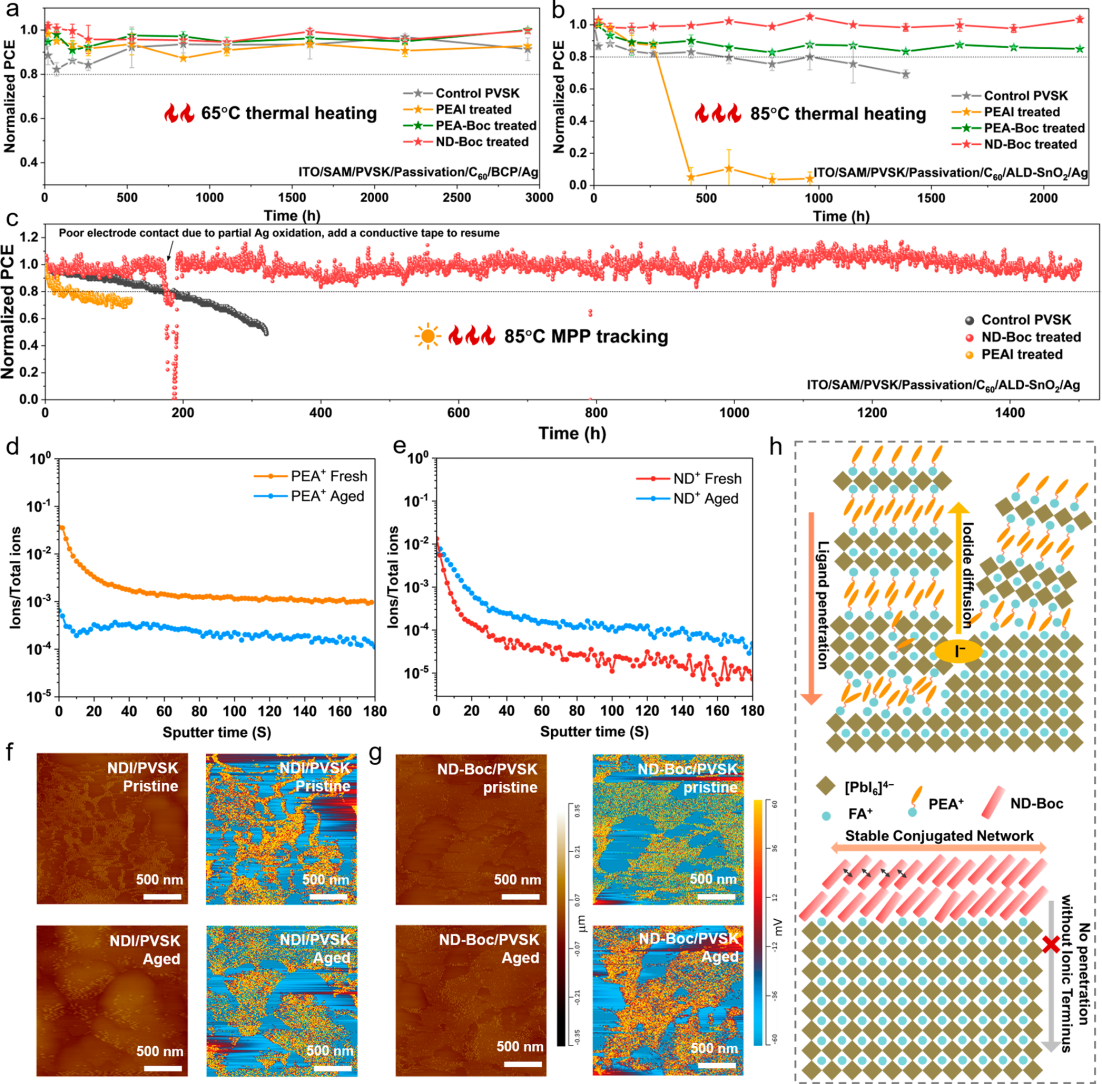
(a) 65 °C thermal stability of PSCs. (b) 85 °C thermal stability of PSCs. (c) MPPT stability at 85 °C under one-sun AM 1.5G illumination using a solar simulator LED array. (d) ToF-SIMS profile of PEAI-modified perovskite films before and after thermal aging at 85 °C. (e) ToF-SIMS profile of ND-Boc-modified perovskite films before and after thermal aging at 85 °C. (f) AFM-IR of NDI-treated perovskite films before (top) and after (bottom) 150 h of thermal aging at 85 °C. Left: topography image; Right: AFM-IR absorption mappings. (g) AFM-IR of ND-Boc-treated perovskite films before (top) and after (bottom) 150 h of thermal aging at 85 °C. (h) Schematic illustration correlating various surface treatments and PSC stability.

Furthermore, all PSCs were encapsulated with cover glass using epoxy UV glue and placed in a customized holder with continuous argon flow for operational stability tracking at 85 °C (Figure 4c). It is important to note that the oxidation of the Ag electrode will also be accelerated when the temperature increases to 85 °C in the customized holder, where trace amounts of oxygen are still present. Thus, a conductive tape was applied to the exposed/partially oxidized Ag electrode to ensure proper connection. The ND-Boc passivated PSC exhibited remarkable MPPT stability at 85 °C under continuous one-sun illumination (corresponding to the ISOS-L-2I level accelerated aging). After 1500 h of MPP tracking, the PSC maintained 96.7% of its initial PCE, comparable to other state-of-the-art work.^40,48–50^ In contrast, control PSCs experienced a significant PCE decay to 48.6% after just 321 h, and PEAI-based PSCs retained less than 80% of their initial PCE after just 30 h of operation. PSCs utilizing the ionic version of ND-Boc (NDI, molecular structure, synthetic route, and device data in Figures S23–S25) exhibited a PCE decline to 58.5% after 153 h. According to the above stability observations, we hypothesized that PEAI with ionic terminus generates PEA-based 2D and quasi-2D perovskite overlayers, which could be a relatively stable protecting layer at mild heating temperatures like 65 °C; however, when subjected to greater thermal stress at 85 °C, the PEA^+^ cations start to diffuse into perovskite lattice by forming quasi-2D perovskites with higher n-value, causing the structural collapse of the 3D bulk perovskite, allowing more severe ion diffusion including iodide diffusion across the layers (Figure 4h). In contrast, ND-Boc is a charge-neutral molecule with no ionic terminus due to Boc substitution, thus unlikely to diffuse into the metal halide lattice of perovskite. Moreover, ND-Boc layers, featuring a conjugated naphthalimide-based network with π-π stacking and multiple H-bonding, can serve as stable hydrophobic overlayers to suppress ion diffusion due to their steric bulk and strong intermolecular interactions. Regarding the deprotonation of FAI^34^ and consequent de-Boc reaction, NMR characterization confirms that no reaction occurs between FAI and ND-Boc (Figure S26). Trace acid generation from FAI and slow solid-state kinetics make the de-Boc reaction unlikely.

To gain insight into PSC stability and confirm our hypothesis, we applied ToF-SIMS to explore the cation penetration in perovskite films (Figure 4d, 4e, and S27–28). Following thermal aging, the intensity gradient of the PEA cation on the perovskite surface diminishes, accompanied by the emergence of a new bump located between 20 and 60 s of sputter time, indicating a deeper penetration into the perovskite films. In contrast, for the ND-Boc sample, the gradient decay remains significant, with the majority of the ND-Boc still concentrated on the surface and only a slight increase in concentration observed within the perovskite films. The observed signal enhancement could also arise from fluctuations in ion intensity during mass spectrometric measurements. We have also fabricated full perovskite devices for ToF-SIMS measurements (Figure S29). Comparing PSCs before and after 150 h of 85 °C thermal aging, the iodide (I^–^) and silver (Ag^+^) ion concentrations increased at the upper layers of perovskites, mainly from the middle perovskite layers and upper electrodes, respectively. In contrast, PSCs with ND-Boc passivation exhibited no increase in the I^–^ and Ag^+^ ion concentration at the upper surface of perovskite, revealing much better stability. To characterize the planar distribution of passivators, AFM-IR was employed to compare ND-Boc with ionic NDI, as both have C=O signals from the naphthalimide unit that can be identified by IR (Figure 4f and 4g). We observe that NDI preferentially wraps around perovskite grain boundaries by correlating the topography (left) with AFM-IR absorption mapping (right) of fresh NDI/PVSK films. This is attributed to its ability to bind more readily to excess PbI_2_ at the perovskite boundaries in the form of ammonium salt. After aging, NDI expands and diffuses into the surface of the grains. In contrast, ND-Boc, as a neutral molecule, exhibits relatively weaker interactions with PbI_2_, requiring a longer duration to adhere to the surface of the perovskites. It consistently covers the surface of the grains both before and after thermal aging, demonstrating enhanced lateral stability. By integrating the results from 65 and 85 °C thermal stability tests (ISOS-D-2I) and 85 °C MPPT stability tests (ISOS-L-2I), it can be conclusively stated that for interfacial passivation, 2D perovskite layers formed with ionic passivators, represented by PEAI, tend to experience ion diffusion at 85 °C elevated temperature, which is detrimental to the stability of PSCs. Molecular modulation of the ionic terminus, conjugated backbone, and energetics, represented by ND-Boc molecules, plays a vital role in improving the lifetime of PSCs, especially under challenging aging conditions with substantial thermal and photo stress.

We further monitored changes in perovskite film morphology before and after 90 h of heating at 65 °C under 1-sun illumination using SEM (Figure 5a-5d). For the control and PEAI samples, the surface developed more holes due to the evaporation of volatile species, resulting in a more amorphous and less crystalline surface. PEA-Boc retained most of the large crystal domains but still exhibited slightly increased surface roughness. In contrast, ND-Boc preserved most of its crystalline structure. These results were also well-supported by AFM measurements (FiguresS13 and S30). The root-mean-square (RMS) roughness for the control sample increased notably from 18.2 to 27.6 nm. The PEAI sample also exhibited an increased roughness, changing from 22.5 to 26.7 nm. In the case of PEA-Boc, the roughness rose from 18.2 to 21.8 nm. Conversely, the ND-Boc sample showed almost no change in surface roughness, with values remaining relatively stable from 19.8 to 19.6 nm. Further, surface potential mapping and statistical distribution were obtained using KPFM (Figures 5e–5l). After 90 h of aging at 65 °C under 1-sun illumination, more PbI_2_ and iodide vacancies were produced, making the surface more n-type with increased surface potential. For the control sample, the surface potential increased from −397 mV to −220 mV. PEAI and PEA-Boc also showed increases from −301 mV to −208 mV and −354 mV to −215 mV, respectively. Moreover, the distribution for PEAI is broadened. In contrast, the surface potential exhibited only a 21 mV increase for ND-Boc, from 314 mV to 335 mV. The intact surface morphology, consistent surface roughness, and stable surface potential collectively contributed to the superior stability of ND-Boc-based PSCs compared to other materials.

**Figure 5:**
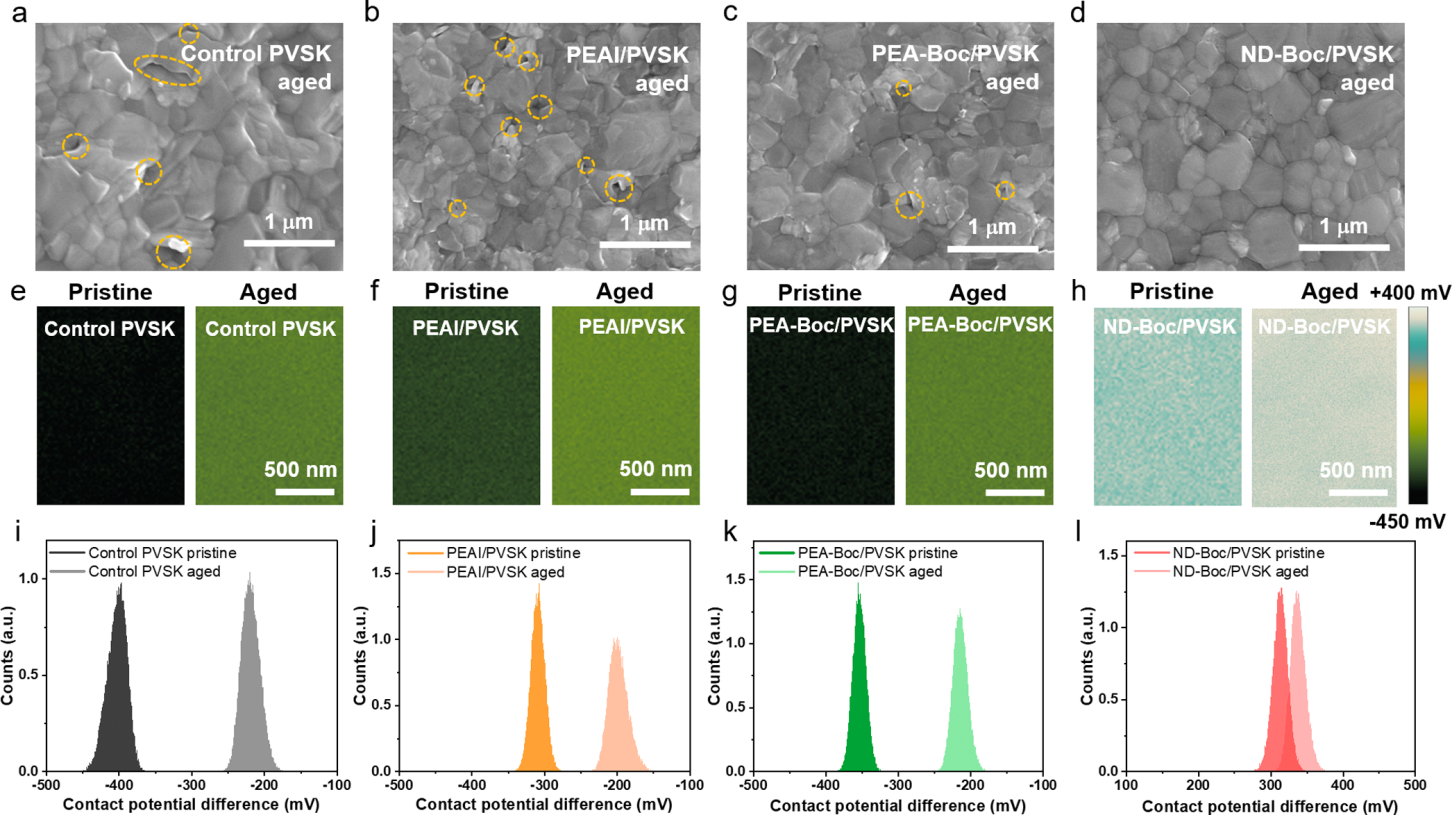
SEM of aged (a) perovskite films without passivation, (b) PEAI passivated perovskite films, (c) PEA-Boc passivated perovskite films, and (d) ND-Boc passivated perovskite films. The aging condition is continuous 65 °C thermal heating and 1-sun light exposure in a N_2_ environment for 90 h. (e–h) Kelvin probe force microscopy (KPFM) with a surface potential change of perovskite films with different surface treatments before and after 90 h of 65 °C thermal heating and 1-sun light exposure in a N_2_ environment. (i–l) Statistical potential distribution of perovskite films with different surface treatments before and after 90 h of 65 °C thermal heating and 1-sun light exposure in a N_2_ environment.

## Conclusion

To improve the limited high-temperature thermal stability of PSCs with ionic passivators, we employed a molecular-engineered ND-Boc to convert commonly used ionic passivators into charge-neutral molecules for interfacial passivation. The extended conjugation and introduced electron-accepting moieties of ND-Boc can further enhance the efficiency of PSCs. More importantly, the ion diffusion across interfaces can be suppressed and the long-term stability of PSCs can be significantly improved. With the passivation of the new ND-Boc molecule, PSCs and 5 cm × 5 cm mini-modules demonstrated a champion efficiency of 26.04% and 23.63% (aperture-area PCE of 21.83%), respectively. The unencapsulated ND-Boc-based PSCs reveal excellent thermal stability at 65 and 85 °C. More impressively, the ND-Boc passivated PSC retained 96.7% of its original efficiency after MPP tracking at 85 °C for 1500 h (ISOS-L-2I protocol). This simple and straightforward Boc substitution method can be generally applicable to fabricate highly efficient and scalable PSCs with significantly improved stability to pave the way for industrial applications.

## Supplementary Material



## Notes

Materials synthesis, device characterization, and stability measurements (PDF)
