# Supplementary material for: Molecular Engineering of Terminus, Conjugation, and Energetics for Thermally Stable Inverted Perovskite Solar Cells
Source: J Am Chem Soc. 2025 Aug 22;147(35):31965–74. doi: 10.1021/jacs.5c09669 (PMC12755201; doi:10.1021/jacs.5c09669)
Supplement: Supplementary file 1 [file ja5c09669_si_001.pdf]

Supporting Information

Molecular Engineering of Terminus, Conjugation, and Energetics for Thermally Stable Inverted Perovskite Solar Cells

Jiaonan Sun[†,a,c,d], Jiarong Wang[†,a,d], Ze-Fan Yao[e], Leyu Bi[b,d], Xiaofei Ji[f], Jia Wang[g], Xiaofeng Huang[a,d], Ming Liu[a,d], Kaikai Liu[a,d], Francis R. Lin[b,d], Bin Kan[g], Qiang Fu*[a,d] , Alex K.-Y. Jen*[a,b,d,h]

[a]Department of Materials Science and Engineering, City University of Hong Kong, Kowloon, Hong Kong 999077, China

[b]Department of Chemistry, City University of Hong Kong, Kowloon, Hong Kong 999077, China

[c]School of Chemical Engineering and Light Industry, Guangdong University of Technology, Guangzhou 510006, China

[d]Hong Kong Institute for Clean Energy, City University of Hong Kong, Kowloon, Hong Kong 999077, China

[e]College of Chemistry and Molecular Engineering, Peking University, Beijing 100871, China

[f]The Interdisciplinary Research Center, Shanghai Advanced Research Institute, Chinese Academy of Sciences, Pudong, Shanghai 201210, China

[g]School of Materials Science and Engineering, National Institute for Advanced Materials, Nankai University, Tianjin 300350, China

[h]State Key Laboratory of Marine Pollution, City University of Hong Kong, Kowloon, Hong Kong 999077, China

[†]These authors contributed equally to this work

*Corresponding author. Email: qfu222@cityu.edu.hk (Q. Fu); alexjen@cityu.edu.hk (A.K.Y.J.)

1. Materials and synthesis

All materials were used as received without further purification. Methylammonium chloride (MACl) and formamidinium iodide (FAI) were purchased from Greatcell Solar (Australia). Lead iodide (PbI_2) and hydriodic acid (HI) were purchased from TCI (Japan). Cesium iodide (CsI), lead chloride ($PbCl_2$), fullerene 60 (C_{60}) and bathocuproine (BCP, purity of 99.9%) were purchased from Xi'an Yuri Solar Co., Ltd. (China). Solvents, including N,N-dimethylformamide (DMF), dimethyl sulfoxide (DMSO), isopropanol (IPA) and chlorobenzene (CB) were purchased from J&K (China). Silver pellets (Ag) were purchased from Zhongnuoxincai Inc. with a purity of 99.999%. 2,3-Naphthalenedicarboxylic anhydride and N-Boc-ethylenediamine were purchased from Leyan & Co Ltd. CbzNaph was synthesized following the reported literature.[1]

1.1. Synthesis of PEA-Boc, ND-Boc and NDI.

PEA-Boc synthesis: PEAI (2.8 mmol) was dissolved in water and concentrated KOH was added for neutralization. After the aqueous solution turned cloudy, dichloromethane (DCM) was used to extract the organic phenethylamine 2 times. The collected DCM was dried with magnesium sulfate. After filtration, the obtained solution was directly used for the Boc reaction. Triethyl amine (3.9 mmol) and di-tert-butyl dicarbonate (3 mmol) were added to the phenethylamine. The mixture was stirred at room temperature overnight. DCM and water were added to quench the reaction, and DCM was used to extract the organic phase 3 times. After drying, the solvent was removed by rotary evaporation. For purification, hexane was used to dissolve the

product, and then placed the solution in the freezer. Solid precipitate (PEA-Boc) was collected in ca. 70% yield. ^{1}H NMR (400M Hz, $CDCl_3$). δ 7.31 (t, J = 7.4 Hz, 2H), 7.25 – 7.16 (m, 3H), 3.38 (q, J = 6.8 Hz, 2H), 2.80 (t, J = 7.1 Hz, 2H), 1.43 (s, 9H).

ND-Boc synthesis: 2,3-Naphthalenedicarboxylic anhydride (1.43 mmol), N-Boc-ethylenediamine (1.58 mmol) and 38 ml DMF were added into a 250 ml round-bottom flask. The mixture is stirred at 120 °C for 3 hours. Water was used to quench the reaction and ethyl acetate was used to extract the organic phase. By rotary evaporation, the solvent was removed, and the crude product was further purified using column chromatography (DCM and ethyl acetate). 420 mg white powder (ND-Boc) is obtained after purification. ^{1}H NMR (400 MHz, $CDCl_3$) δ 8.35 (s, 2H), 8.06 (dd, J = 6.1, 3.3 Hz, 2H), 7.70 (dd, J = 6.2, 3.3 Hz, 2H), 4.89 (s, 1H), 3.90 (m, 2H), 3.48 (d, J = 5.3 Hz, 2H), 1.34 (s, 9H).

NDI synthesis: In a 50 ml round-bottom flask, ND-Boc (0.15 mmol) was mixed with HI (57wt% in water, 0.30 mmol) and 25 ml ethanol. The reaction mixture was further heated to reflux. After 3 hours, the reaction was completed, as monitored by TLC. The mixture was dried by rotary evaporation, and the solid precipitated upon the addition of diethyl ether. The solid was filtered and washed with copious amounts of diethyl ether, then dried under a vacuum. In the end, white powder (NDI) was obtained in a quantitative yield. ^{1}H NMR (400M Hz, DMSO-d6) of NDI. δ 8.57 (s, 2H), 8.29 (dd, J = 6.2, 3.4 Hz, 2H), 7.80 (dt, J = 6.7, 3.4 Hz, 5H), 3.90 (t, J = 5.9 Hz, 2H), 3.13 (t, J = 5.9 Hz, 2H).

1.2 Synthesis of PEA-Boc and ND-Boc Single Crystal

The PEA-Boc single crystals were grown by solvent evaporation. PEA-Boc was dissolved in hexane and then placed in the fridge. After a few weeks, needle-like crystals were obtained. The ND-Boc single crystals were also grown by solvent evaporation. ND-Boc was first dissolved in IPA by thermal heating and then placed in the air at room temperature. After a few days, needle-like crystals were obtained.

1.3 Perovskite film preparation and device fabrication (Small-area)

ITO glass (15 Ω sq^{-1}; 1.5 × 1.5 cm^2 for a small-area device) was progressively washed by sonication with detergent (Decon 90), deionized water, acetone and IPA for 15 min. The washed ITO glass was dried in a 75 °C oven for over 12 hours. Then, the cleaned ITO substrates were subjected to ultraviolet ozone for 30 min and transformed into a N_2-filled glovebox for film fabrication. CbzNaph (1.5 mg mL^{-1} in IPA) was spin-coated onto the cleaned ITO at 3,000 rpm for 30 s, followed by annealing at 100 °C for 10 min. The substrates were cooled to room temperature, washed with pure IPA, and then annealed at 100 °C for 10 min. For the perovskite precursor solution, 1.33 M FAI, 1.47 M PbI_2, and 0.07 M CsI were dissolved in 1 mL DMF: DMSO mixed solvent (4:1 v:v). Besides, 3% $PbCl_2$ and 10% MACl were added to the precursor solution before spin-coating. 50 µL of the prepared precursor solution was spin-coated at 2000 rpm for 10 s and 5,000 rpm for 50 s onto the CbzNaph-based ITO substrate; 170 µL CB as the antisolvent was dripped on the film at 5 s before the end of the last procedure and then annealed at 100 °C for 30 min. As for the passivation layer, PEAI, PEA-Boc or ND-Boc (1 mg/mL in IPA) is spin-coated on perovskite film at 3000 rpm for 30 s, then annealed at 100 °C for 10 min. The films were then cooled to room temperature and

readied for thermal evaporation. 25 nm C_{60}, 6 nm BCP, and 100 nm Ag were sequentially evaporated onto the substrate under a high vacuum (<4 × 10^{-6} torr). For the anti-reflection coating, a MgF_2 layer with a thickness of 140 nm was thermally evaporated onto the back of the devices. A metal shadow mask with an aperture area of 0.06 cm^2 was used to determine the cell contact area. The perovskite solar cells were covered with a shading mask with an aperture area of 0.04 cm^2 during *J*-*V* scans to ensure the accuracy of current density from *J*-*V* curves.

1.4 Device Fabrication (Mini modules)

First, the ITO substrate (5 × 5 cm^2) was cleaned with detergent, DI water, acetone and ethanol under an ultrasonic bath (30 min for each step). P1 was etched by laser. The high laser power was used to ensure the clean etching of the ITO conducting layer. The perovskite precursor solution for the module was the same as that used on the small-size substrate. Then, 150 µL precursor was spin-coated at 2,000 rpm for 10 s and 5,000 rpm for 80 s onto the CbzNaph-based ITO substrate; 550 µL CB as the antisolvent was dripped on the film at 10 s before the end of the spin procedure and then annealed at 100 °C for 30 min. As for the passivation layer, PEAI, PEA-Boc or ND-Boc (1 mg/mL in IPA) is spin-coated on perovskite film at 3000 rpm for 30 s, then annealed at 100 °C for 10 min. The films were then cooled to room temperature and readied for thermal evaporation. Subsequently, 25 nm C_{60} and 6 nm BCP were sequentially evaporated under a high vacuum (<5 × 10^{-6} torr). After that, P2 was etched by laser. Then, 100 nm Ag was evaporated as the electrode. Finally, P3 was etched by laser. GFF is about 92.4%.

1.5 Atomic layer deposition (ALD) of SnO_2

ALD equipment is purchased from Beijing Antechnology Co., LTD. Tetrakis(dimethylamino)tin(IV) (TDMASn) (at 55 °C) and H_2O (at 25 °C) were used as precursors for SnO_2 growth. Nitrogen as carrier gas and process flow rates was set to 150 sccm. SnO_2 growth consists of a cyclical process of the TDMASn dose (0.35 s), a purge (20 s), a water dose (0.5 s) and a purge (20 s). 200 cycles are needed for the growth of SnO_2 film with a thickness of 30 nm.

2. Measurements

The ^{1}H NMR spectra were conducted with a Bruker 400MHz AVANCE III spectrometer. The XRD patterns for films are measured by X-ray diffraction (XRD) using a Bruker D2 Phaser with Cu Kα radiation. Ultraviolet-visible (UV-vis) absorption spectra were recorded on a UV-Vis spectrometer (PerkinElmer Lambda 1050+ UV/Vis/NIR spectrophotometers with labsphere). The top-view images of the samples were acquired by scanning electron microscopy (SEM, Philips XL30 FEG). The UPS and XPS characterizations were performed by a VG ESCALAB 220i-XL surface analysis system equipped with a He discharge lamp (hv = 21.22 eV) and a monochromatic Al-Kα X-ray gun (hv = 1486.6 eV). The characterized peak of hydrocarbon C1s from adventitious carbon at 284.8 eV was used for binding energy calibration. Atomic force microscope-infrared spectroscopy (AFM-IR) was performed on IR-neaSCOPE^{+S} from Quantum Design. The C=O characteristic peaks from ND-Boc and NDI were detected for surface mapping. A contact angle meter (DataPhysics

Contact Angle Tester) was used to measure the contact angles of water droplets on sample substrates. Thermal gravimetric analysis (TGA) was performed on Mettler Toledo TGA 2 STAR System. ToF-SIMS measurements were performed on a TOF. SIMS5-100. Cs^+ was used as sputtering ions with 1 keV ion energy, 60 nA ion current, and 200 × 200 μm^2 raster size. While analysis was carried out using a pulsed primary ion beam of Bi^{3+} (30 keV). An area of 50 × 50 μm^2 was measured. The trap-state density of control and target devices were measured by SCLC. The dark current-voltage response of the electron-only devices using a diode configuration of ITO/SnO_2/perovskite/C_{60}/BCP/Ag. The trap-state density is calculated according to the equation. $N_t = \frac{2\varepsilon_r \varepsilon_0 V_{TFL}}{qL^2}$, where ε_r and ε_0 are the relative dielectric constant and vacuum dielectric constant, respectively, V_{TFL} is the trap-filled limited voltage, L is the film thickness of the active layer (700 nm) and q is the elementary charge (1.6×10^{-19} C).

2.1 Photoluminescence (PL) and In-situ PL measurements

PL and in-situ PL spectra were obtained by a home-made equipment, including an excitation system, fiber system, and detector system. The testing samples are held in a humidity-controlled air box with a fiber system set around the sample. In contrast, the excitation and detection system are set in the ambient environment and connected to the fiber. Excitation system using an excitation laser (315 nm, max = 30 W). Excitation light was introduced to the sample through a fiber. The emitted light from the sample was collected by fiber and introduced to a spectrophotometer (Ocean Optics USB2000). A 550 nm low pass filter is applied in the light pass to the spectrophotometer. The in-

situ PL system was integrated into a hot plate for monitoring the 100 °C annealing process.

2.2 Solar cell characterization

The current density-voltage (*J-V*) characteristics of photovoltaic devices were measured in an N_2-filled glovebox at room temperature using a Keithley 2400 Source Meter under simulated sunlight from a solar simulator (EnliTech, SS-F5, Taiwan). The light intensity was calibrated using a silicon solar cell (with a KG-2 filter) from the National Renewable Energy Laboratory. The *J-V* measurements for the cells were conducted with sweep mode with reverse (from 1.20 V to -0.1 V) scan with a scan step of 20 mV and a time delay of 100 ms. For modules, voltage sweep between 7.5 V to -0.1 V with a step of 0.1 V. The *J-V* tests were conducted in a N_2 glovebox. An EQE measurement system was used for the cell (EnliTech, QE-R, Taiwan).

2.3 Long-term device stability measurement

Thermal stability: The unencapsulated cells were placed on the hot plates in N_2 glove box. The temperature of hot plates was 65 °C and 85 °C for 65 °C thermal stability tests and 85 °C thermal stability tests, respectively. Devices were cooled to room temperature for 85 °C thermal stability tests before the *J-V* measurements. For the MPP tracking of devices under 85 °C, the ALD SnO_x layer is used to replace the conventional BCP layer. The solar cells were encapsulated by a cover glass with UV-epoxy glue, with the Ag electrode partially exposed. Then, the encapsulated cell was placed in a customized chamber for connection and airflow. The solar cells were operated at their MPP at 85 °C while being illuminated by an LED source covering wavelengths from

400 to 1,000 nm with one-sun light intensity. The sample chamber was maintained in ambient air throughout the test with a continuous argon flow.

2.4 Computational method

DFT calculations were performed to understand the molecular and interface structures.[2] The crystal structure of perovskite ($FAPbI_3$) was used to build the PbI_2-terminated or FAI-terminated slabs with a molecule on the surface. A vacuum layer of ~15−20 Å was added to avoid self-interactions. The built systems were then energy minimized using Monkhorst-Pack *k*-point mesh at Gamma until the total energies converged to 0.02 meV per atom and displacements less than 0.002 Å. All these periodic DFT calculations were conducted using the Cambridge Serial Total Energy Package, CASTEP academic 22.11 release.[3] Generalized gradient approximation (GGA) with Perdew-Burke-Ernzerh (PBE) parametrization with Grimme's DFT-D3 correction was used with on-the-fly generation (OTFG) ultrasoft pseudopotentials.[4,5] A Real-space mesh cut-off of 550 eV is used for all CASTEP calculations. The binding energy, $E_{binding}$, was calculated from $E_{binding} = E_{slab} - (E_{perovskite} + E_{molecule})$.

3. Supporting Figures

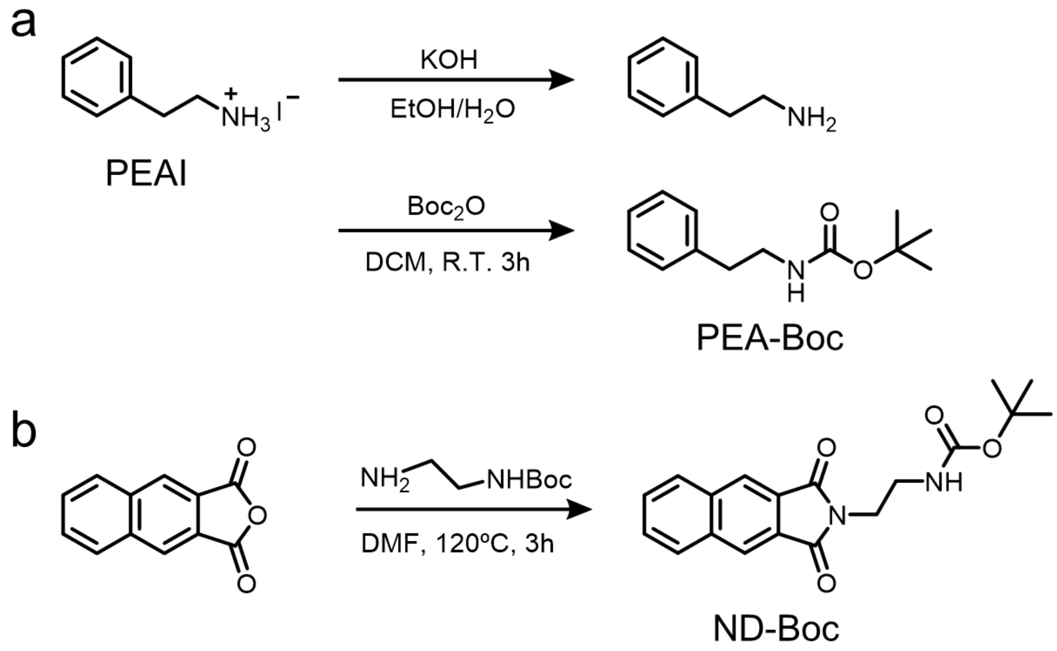

Figure S1. Synthetic route of (a) PEA-Boc and (b) ND-Boc.

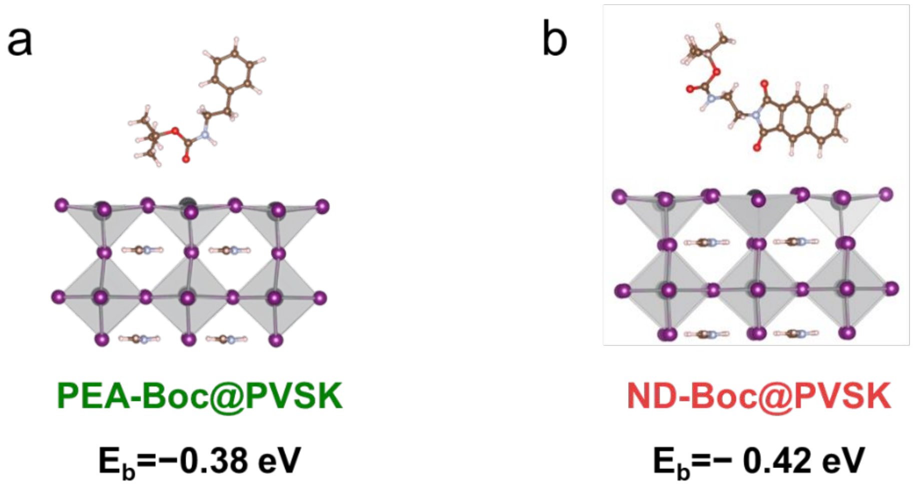

Figure S2. (a) Optimized PEA-Boc with edge-on packing on perovskite PbI_2-rich (100) interface. (b) Optimized ND-Boc with edge-on packing on perovskite PbI_2-rich (100) interface.

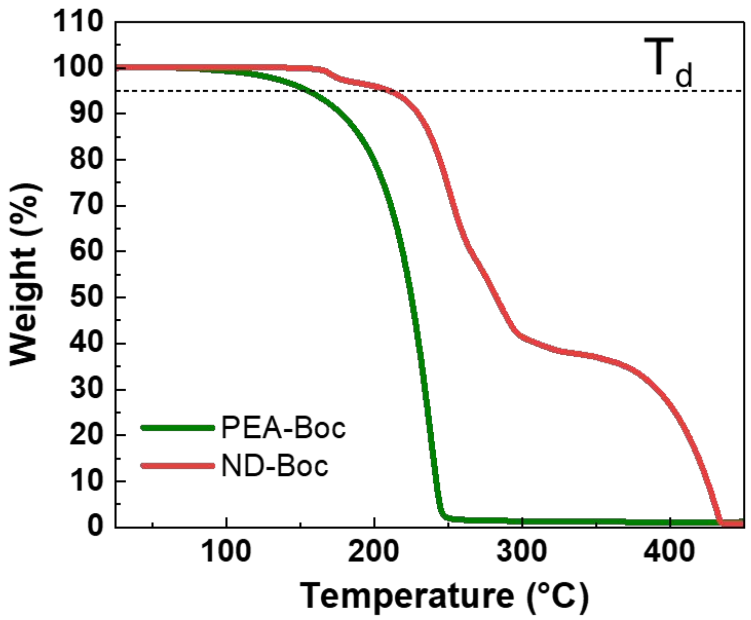

Figure S3. Thermogravimetric analysis (TGA) of PEA-Boc and ND-Boc powders. The decomposition temperature (T_d) for PEA-Boc and ND-Boc is 155 °C and 211 °C, respectively.

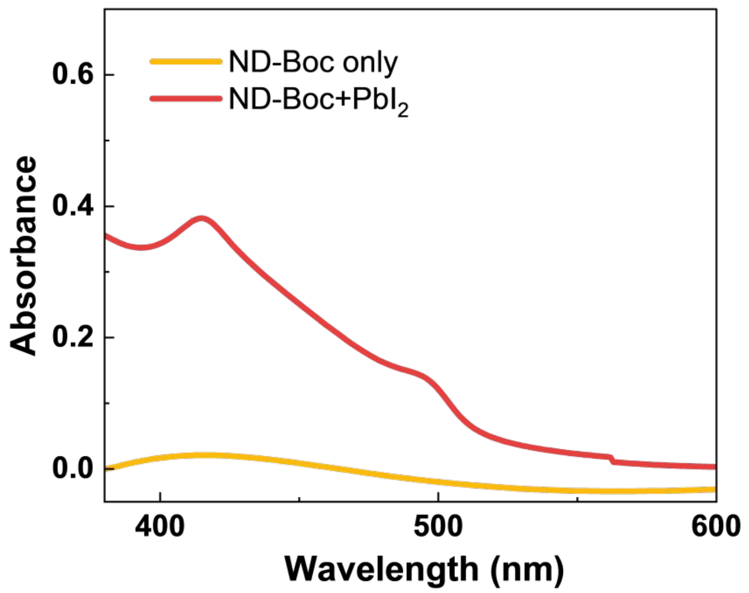

Figure S4. UV-vis of ND-Boc thin films, compared to ND-Boc with PbI_2 addition in 1:1 molar ratio.

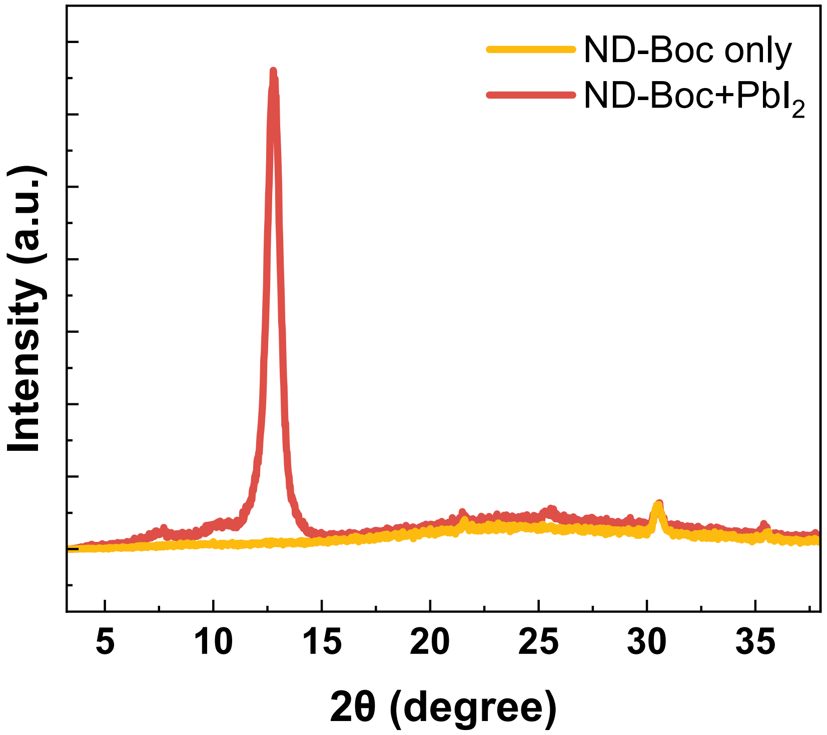

Figure S5. XRD pattern of ND-Boc thin films, compared to ND-Boc with PbI_2 addition in 1:1 molar ratio.

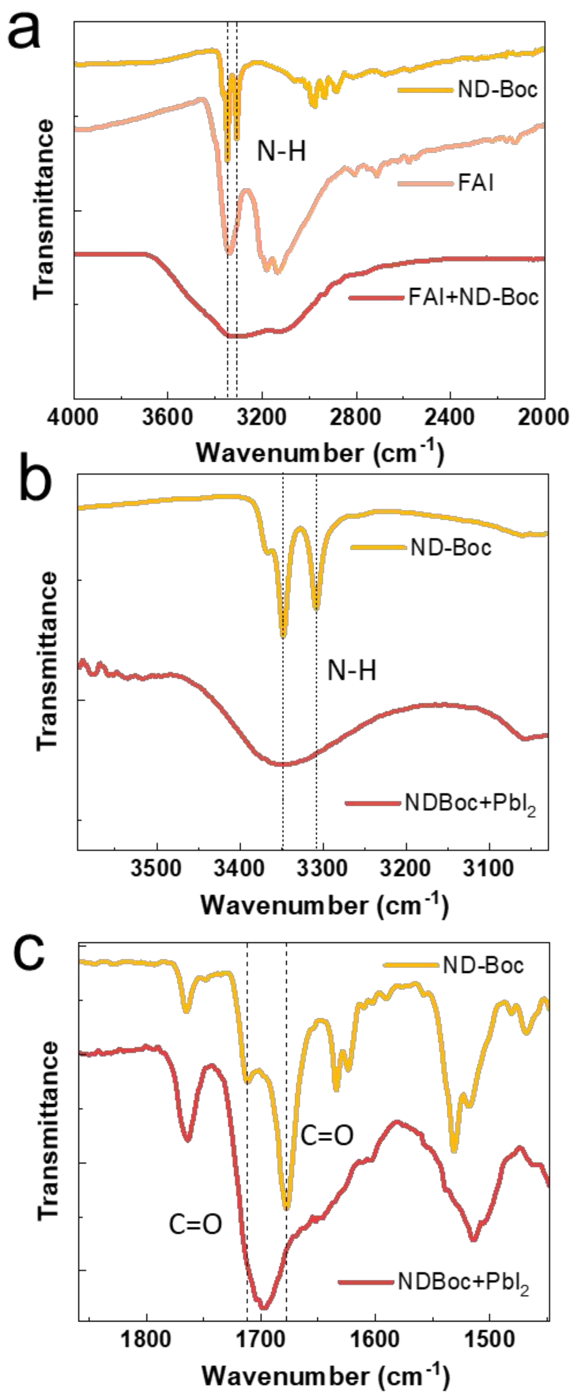

Figure S6. (a) Attenuated total reflectance-Fourier transform infrared spectroscopy (ATR-FTIR) of ND-Boc, FAI and ND-Boc mixed with FAI in a 1:1 molar ratio. (b) ATR-FTIR of ND-Boc and ND-Boc mixed with PbI_2 in a 1:1 molar ratio showing the N-H peaks. (c) ATR-FTIR of ND-Boc and ND-Boc mixed with PbI_2 in a 1:1 molar ratio showing the C=O peaks.

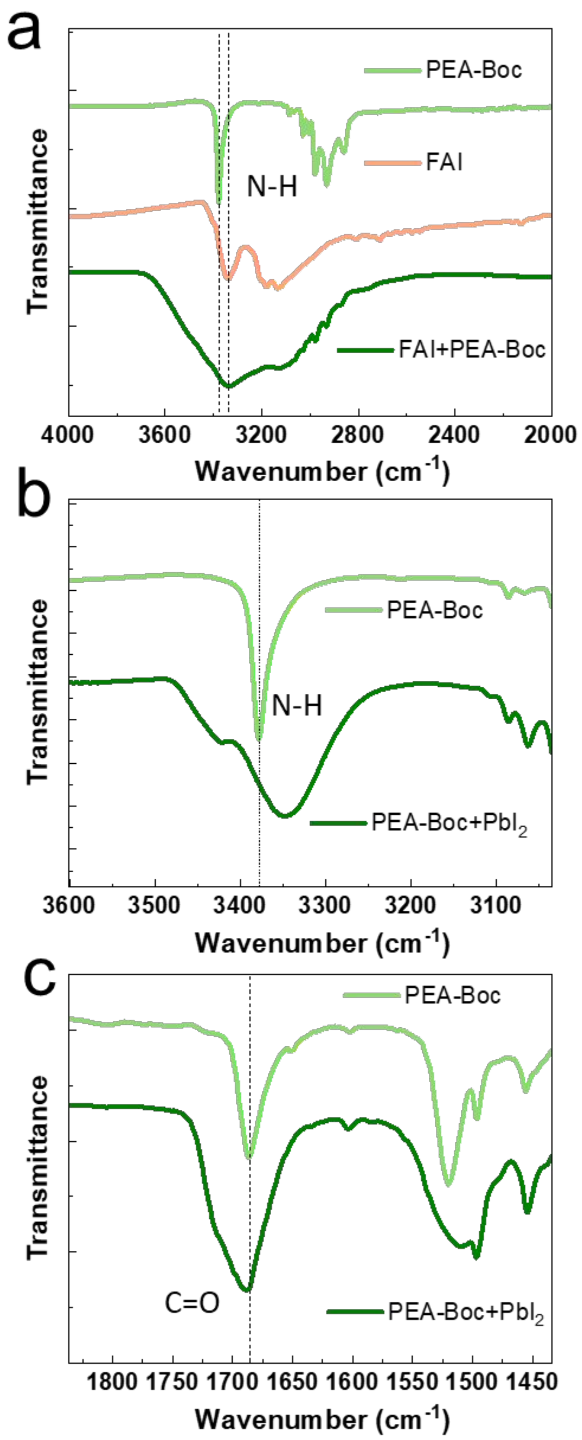

Figure S7. (a) ATR-FTIR of PEA-Boc, FAI and PEA-Boc mixed with FAI in 1:1 molar ratio. (b) ATR-FTIR of PEA-Boc and PEA-Boc mixed with PbI_2 in a 1:1 molar ratio showing the N-H peaks. (c) ATR-FTIR of PEA-Boc and PEA-Boc mixed with PbI_2 in a 1:1 molar ratio showing the C=O peaks.

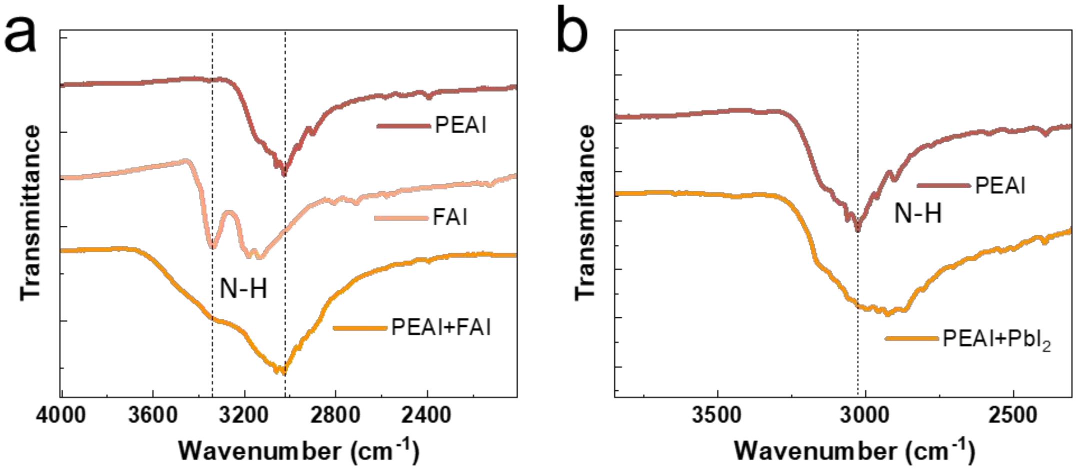

Figure S8. (a) ATR-FTIR of PEAI, FAI and PEAI mixed with FAI in 1:1 molar ratio. (b) ATR-FTIR of PEAI and PEAI mixed with PbI_2 in a 1:1 molar ratio.

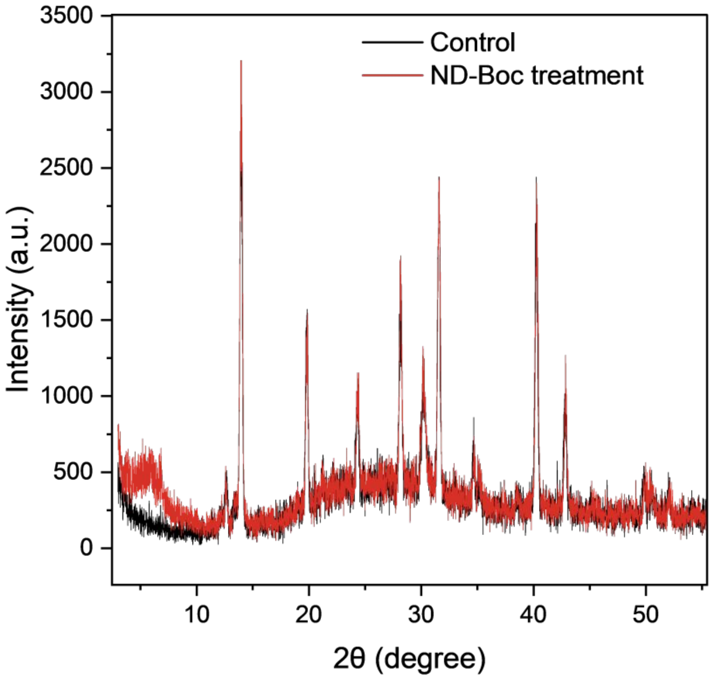

Figure S9. Overlaid XRD patterns of the control and ND-Boc-treated perovskite films (5 mg/ml).

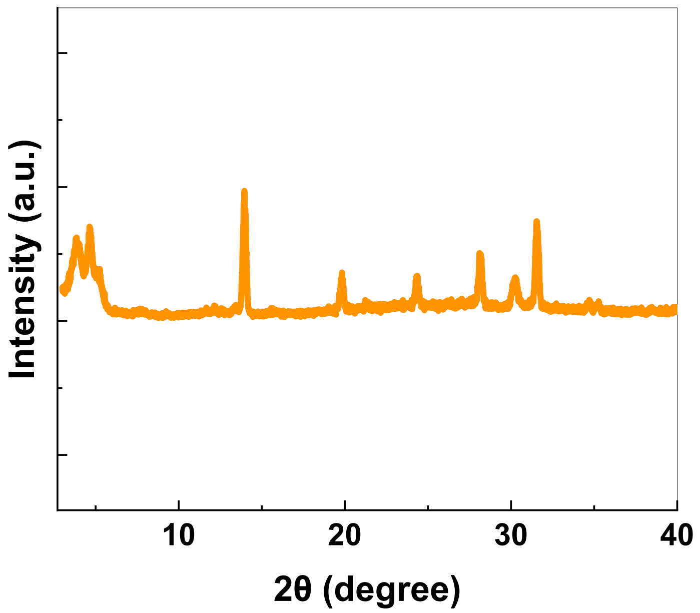

Figure S10. XRD pattern of the 5mg/ml PEAI-treated perovskite film.

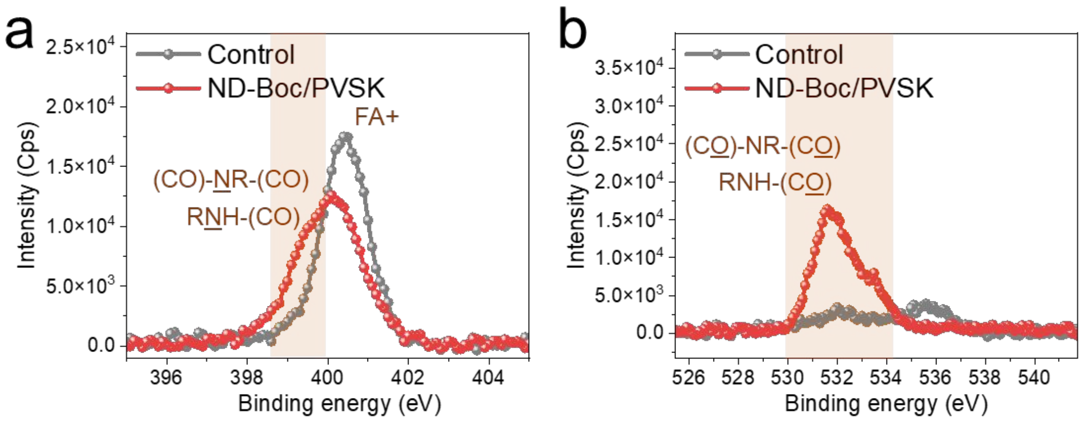

Figure S11. (a) X-ray photoelectron spectroscopy (XPS) spectra of N 1s in control and ND-Boc treated perovskite films. (b) XPS spectra of O 1s in control and ND-Boc treated perovskite films.

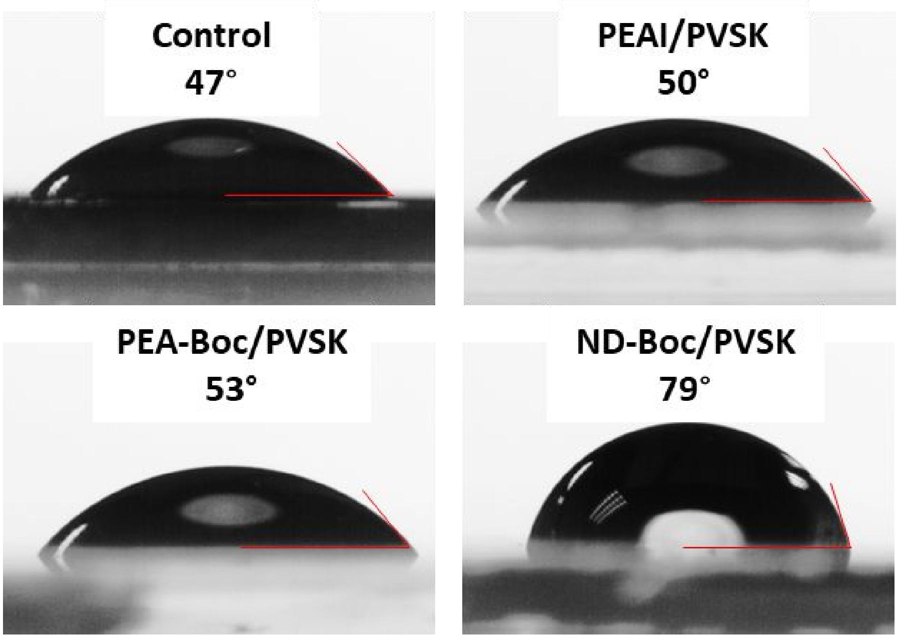

Figure S12. Water contact angles of the control and PEAI/ PEA-Boc/ ND-Boc-treated perovskite films.

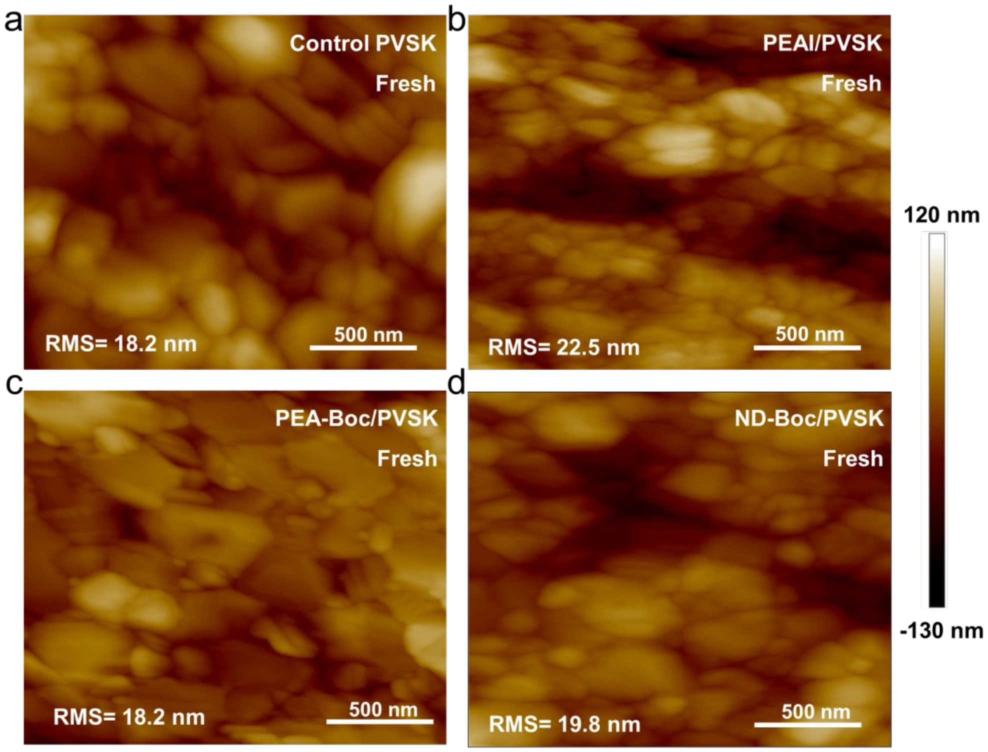

Figure S13. Atomic-force microscopy (AFM) of pristine (a) perovskite films without passivation, (b) PEAI passivated perovskite films, (c) PEA-Boc passivated perovskite films, and (d) ND-Boc passivated perovskite films.

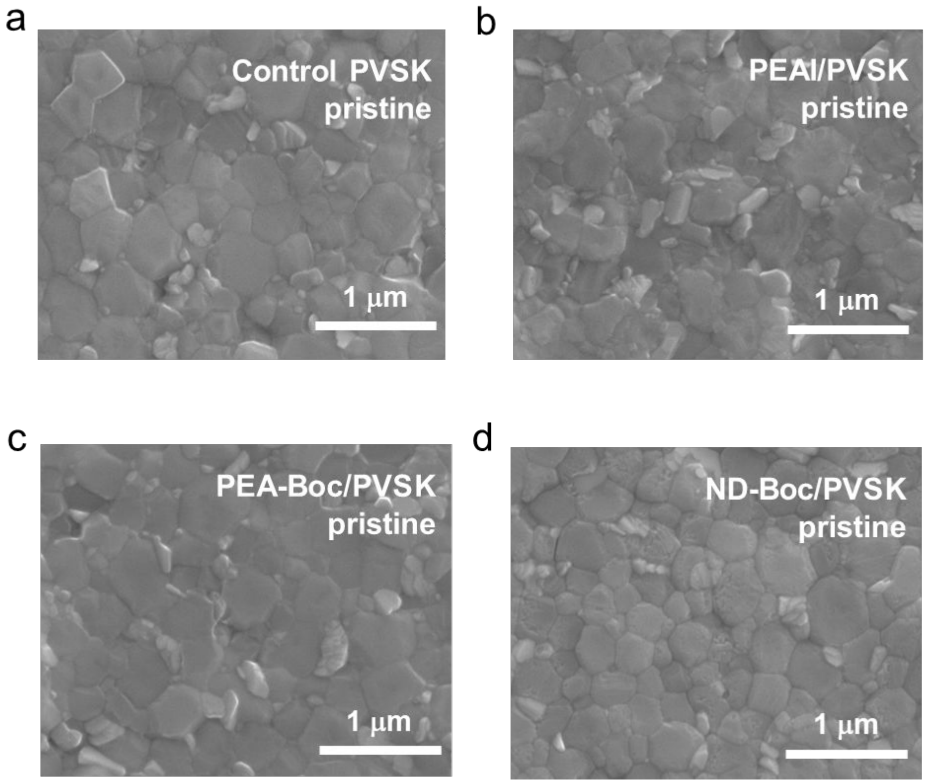

Figure S14. SEM of pristine (a) perovskite films without passivation, (b) PEAI passivated perovskite films, (c) PEA-Boc passivated perovskite films, and (d) ND-Boc passivated perovskite films.

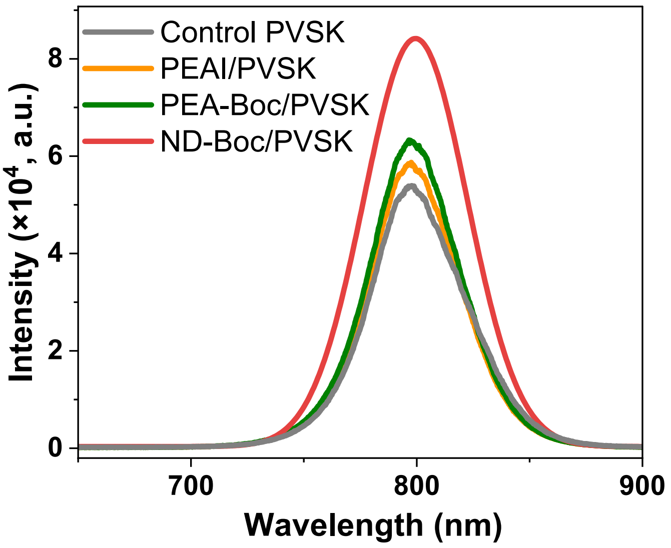

Figure S15. Steady-state PL spectra of control perovskite films, PEAI, PEA-Boc and ND-Boc treated perovskite films.

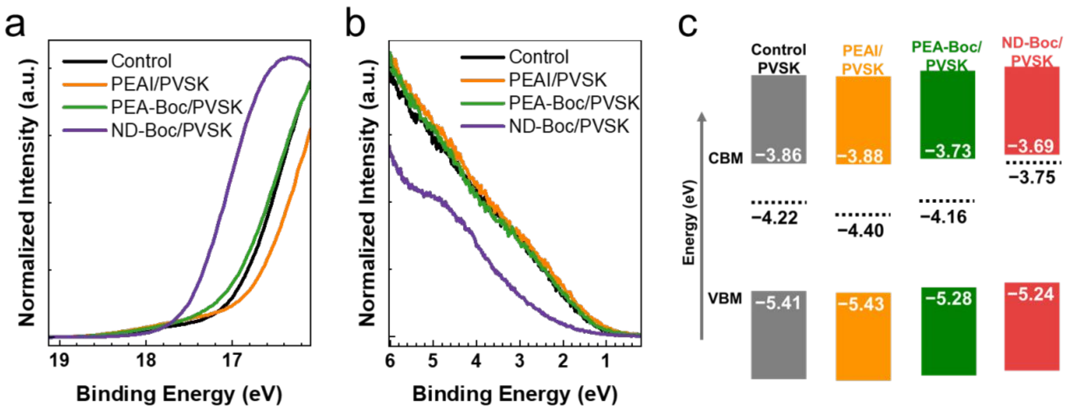

Figure S16. (a) Ultraviolet photoelectron spectroscopy (UPS) spectra of control perovskite films, PEAI, PEA-Boc and ND-Boc treated perovskite films in the cutoff region. (b) UPS spectra of control perovskite films, PEAI, PEA-Boc and ND-Boc treated perovskite films in the onset region (c) Energy alignment extracted from the UPS spectra for the control and PEAI/ PEA-Boc/ ND-Boc-treated perovskite films.

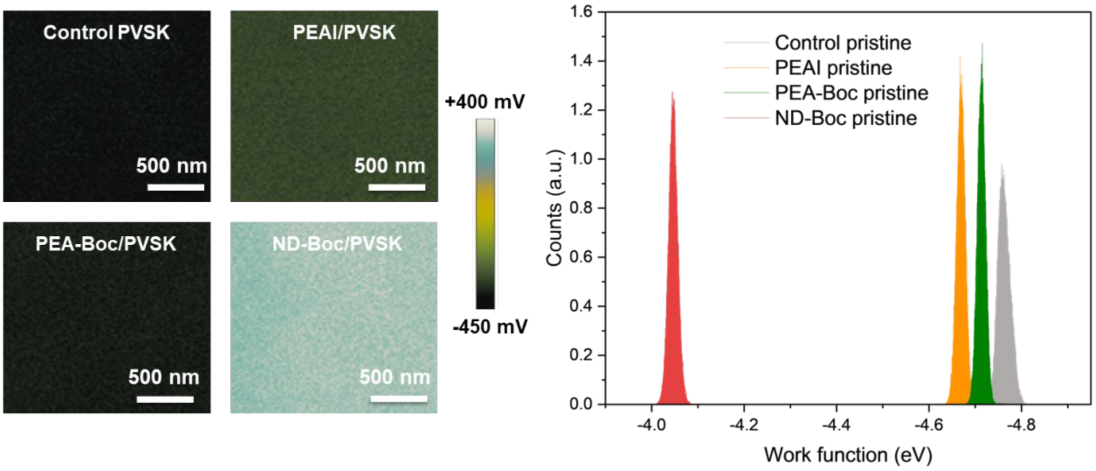

Figure S17. Kelvin probe force microscopy (KPFM) of perovskite films with different surface treatments, work function is calibrated against a Au reference standard.

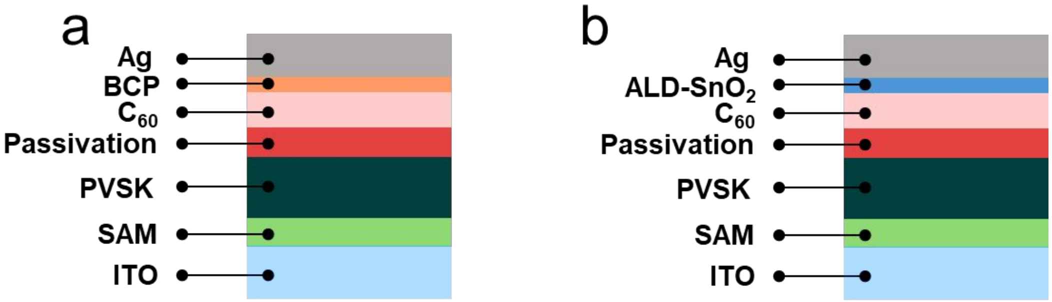

Figure S18. Schematics the inverted PSCs, (a) a standard device architecture. (b) device architecture used for thermal stability and MPPT measurements at 85 °C.

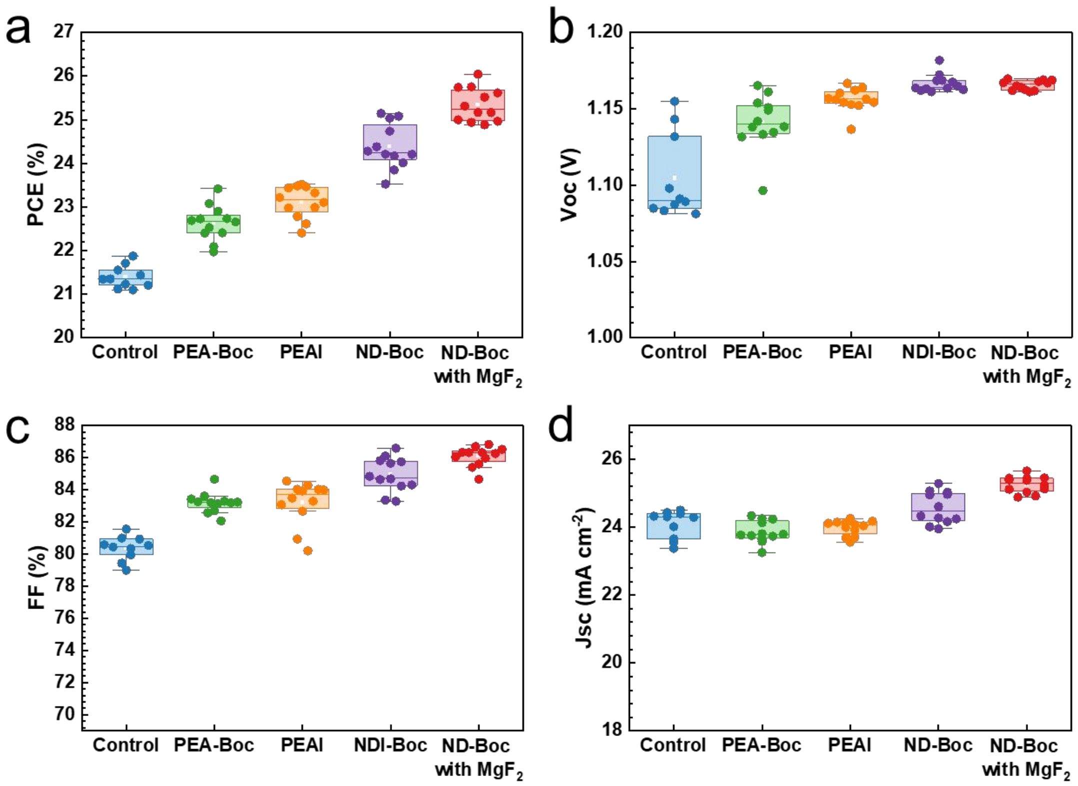

Figure S19. Statistic results showing the (a) PCE, (b) V_{OC}, (c) FF, and (d) J_{SC} distributions of the devices with different passivation (each condition with 12 individual devices). The champion ND-Boc-based devices added a MgF_2 anti-reflection layer to further improve the current and PCE.

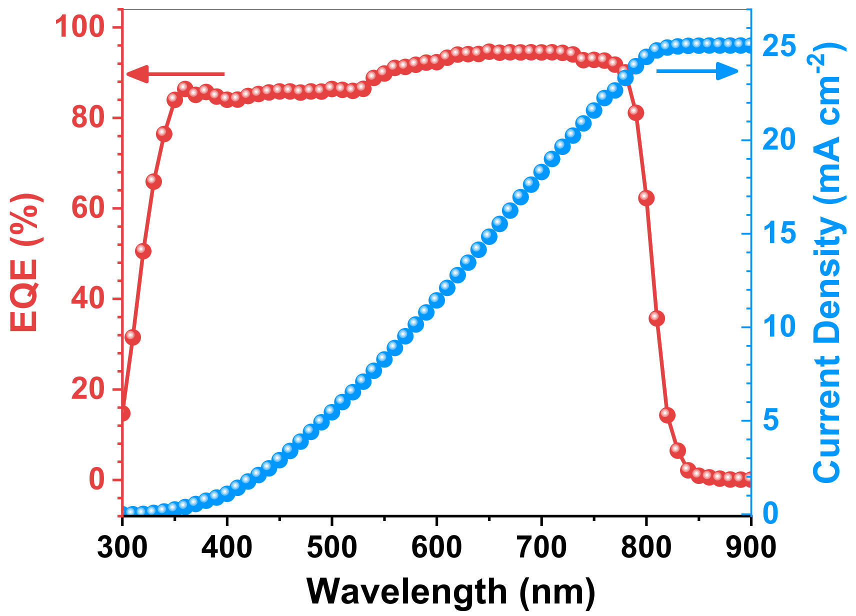

Figure S20. External quantum efficiency (EQE) and integrated J_{SC} of ND-Boc champion device.

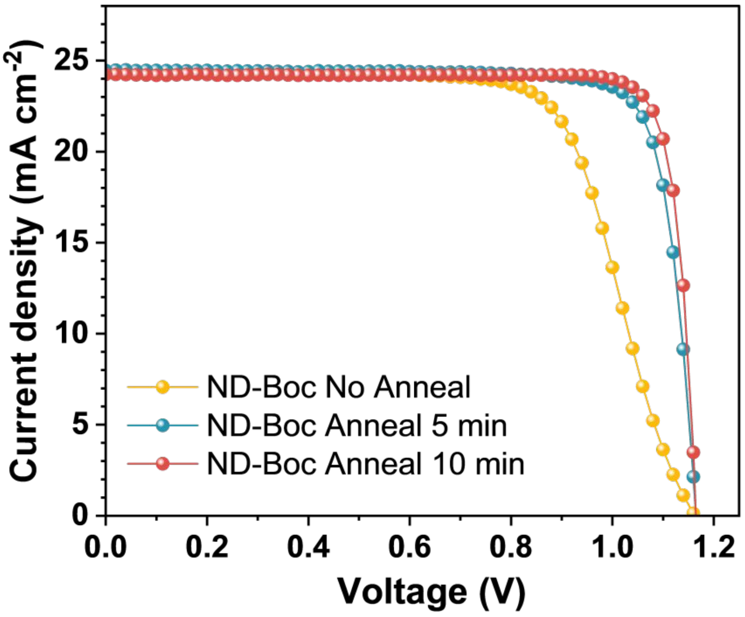

Figure S21. *J–V* curves of ND-Boc based devices without annealing, as well as annealing for 5 min and 10 min. The fill factor increases with prolonged annealing time.

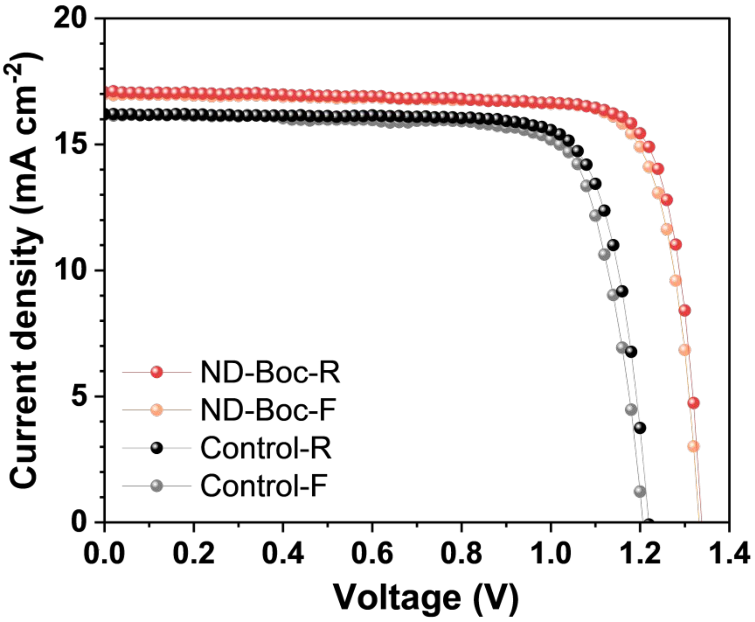

Figure S22. Representative *J–V* curves of the 1.8 eV wide-bandgap pristine and ND-Boc treated PSCs.

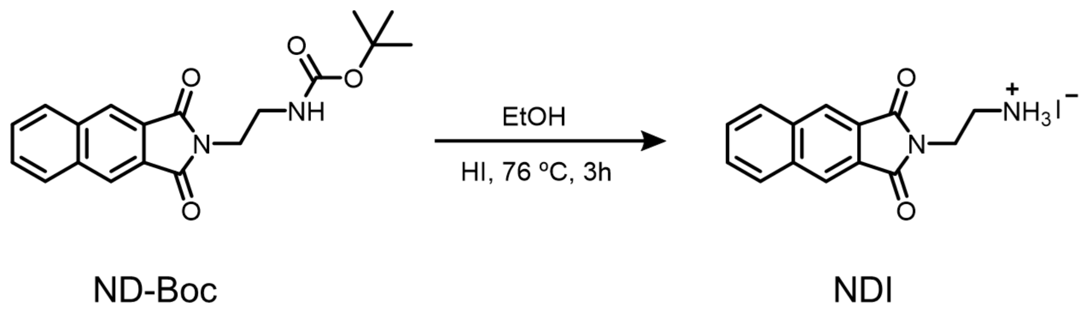

Figure S23. The synthesis of NDI through de-Boc with HI in EtOH solution.

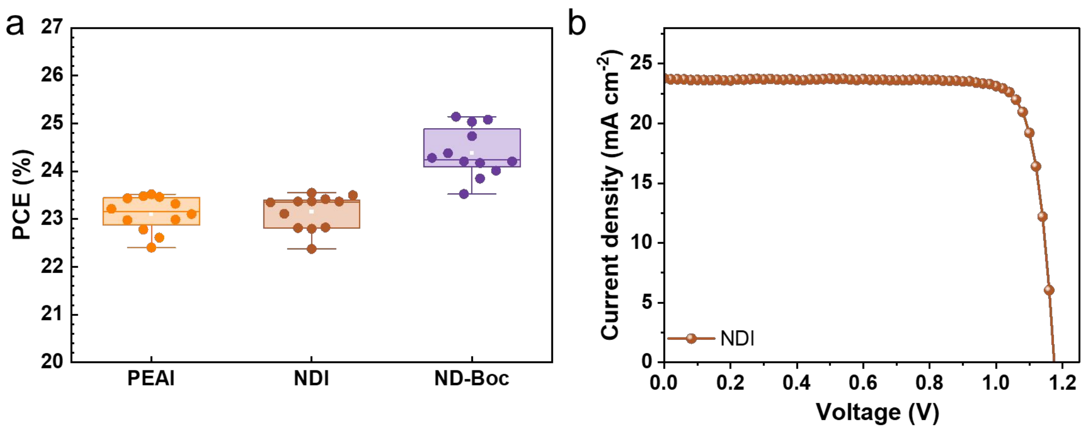

Figure S24. (a) PCE statistic results of NDI-treated-PSCs, in comparison with PEAI and ND-Boc-treated PSCs. (b) The J-V curve NDI-treated-PSCs

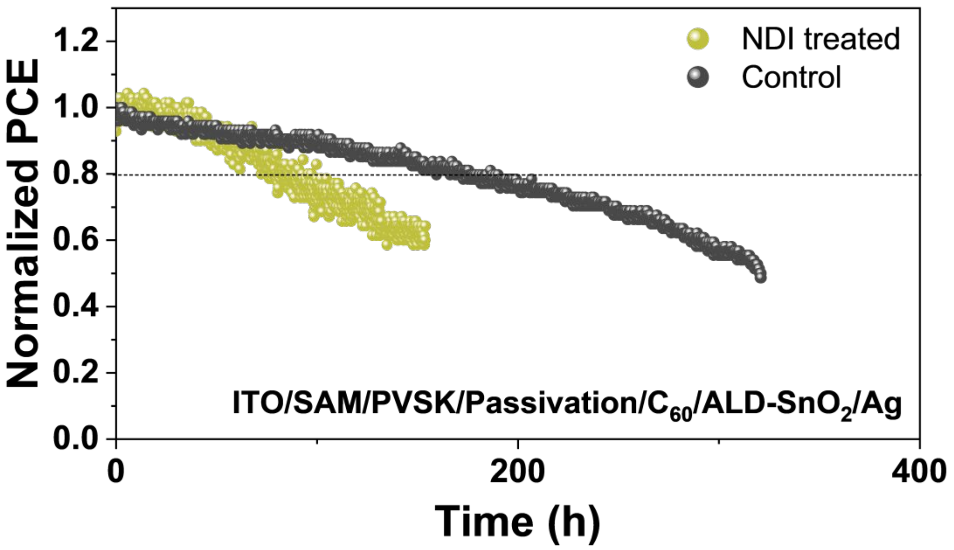

Figure S25. MPPT stability of NDI-treated and control PSCs at 85 °C under one-sun AM 1.5G illumination using a solar simulator LED array.

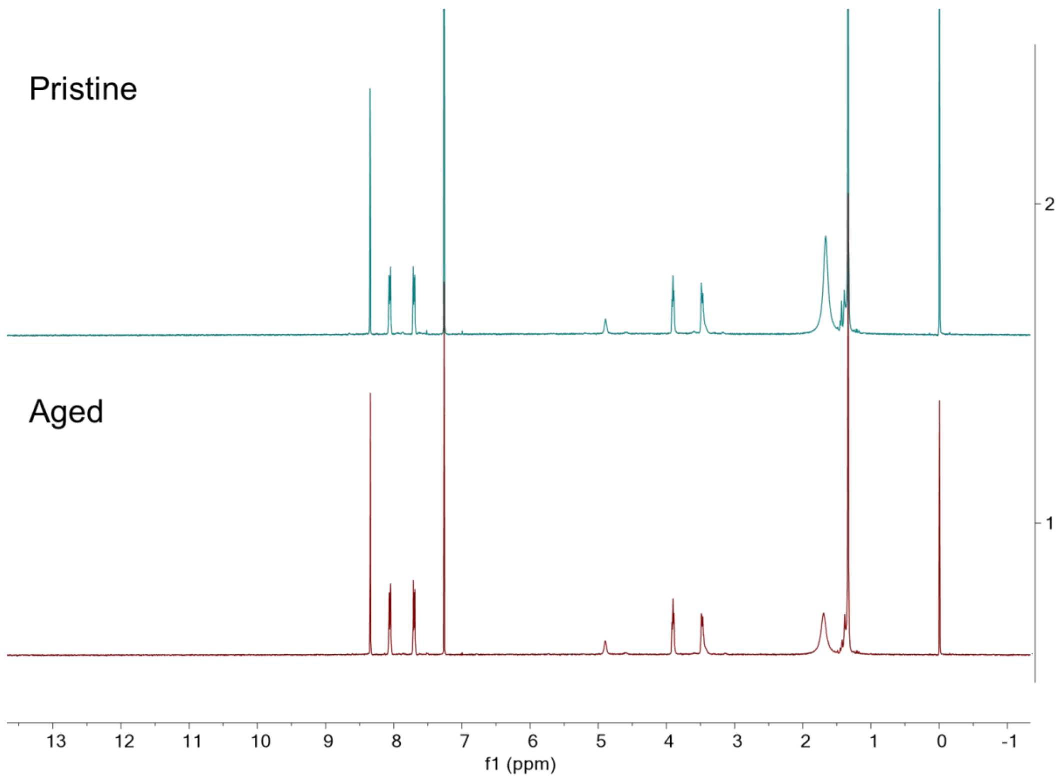

Figure S26. ^{1}H NMR (400MHz, $CDCl_3$) spectra of dissolved bilayer ND-Boc/FAI films. The top spectrum is from the pristine bilayer ND-Boc/FAI films. The bottom spectrum is from bilayer ND-Boc/FAI films after aging at 85°C for 50 hours. The NMR spectra are very similar and show no evidence of ND-Boc degradation.

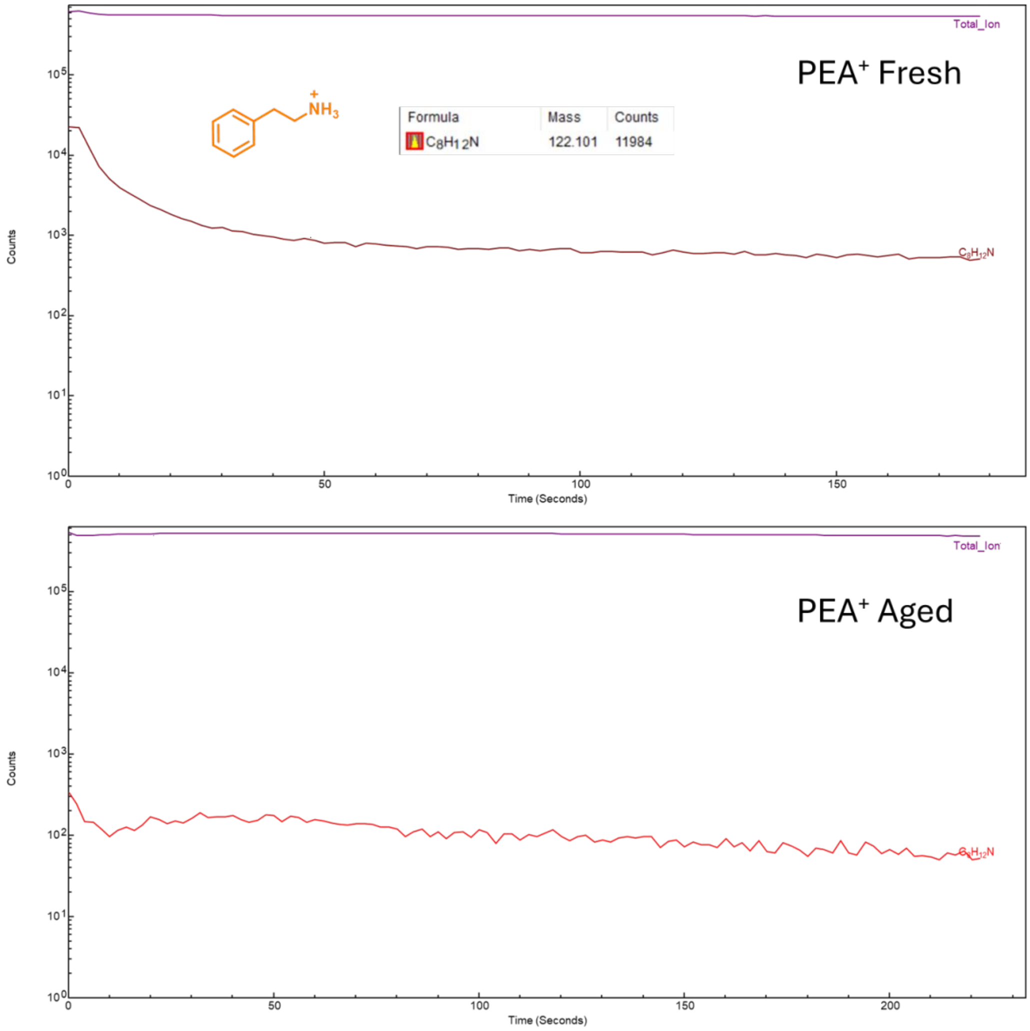

Figure S27. ToF-SIMS profile of PEAI-modified perovskite films before and after thermal aging at 85°C. The ion formular is $C_8H_{12}N^+$ with an exact mass of 122.101 g/mol.

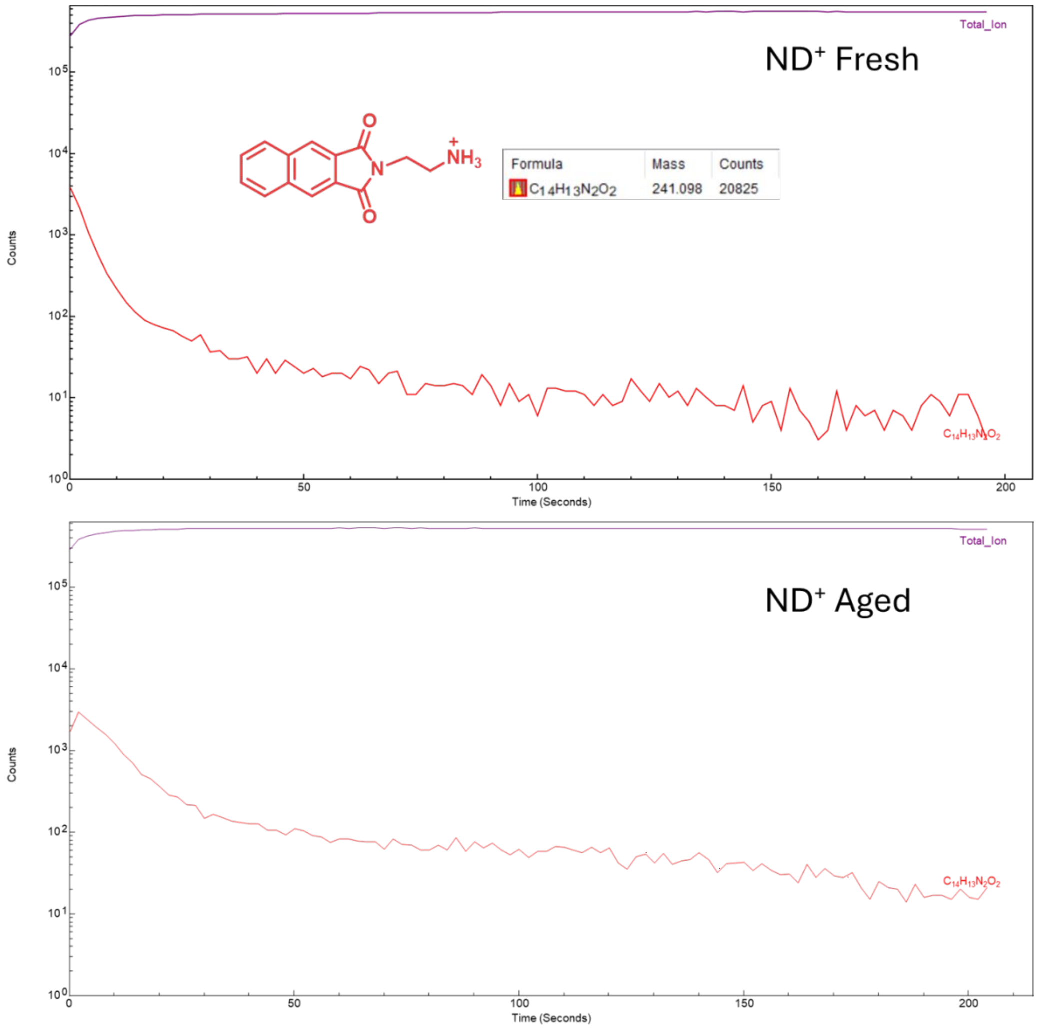

Figure S28. ToF-SIMS profile of ND-Boc-modified perovskite films before and after thermal aging at 85°C. ND-Boc breaks down into ND^+ fragments in the mass spectrum during detection. The ion formular of ND^+ is $C_{14}H_{13}N_2O_2^+$ with an exact mass of 241.098 g/mol.

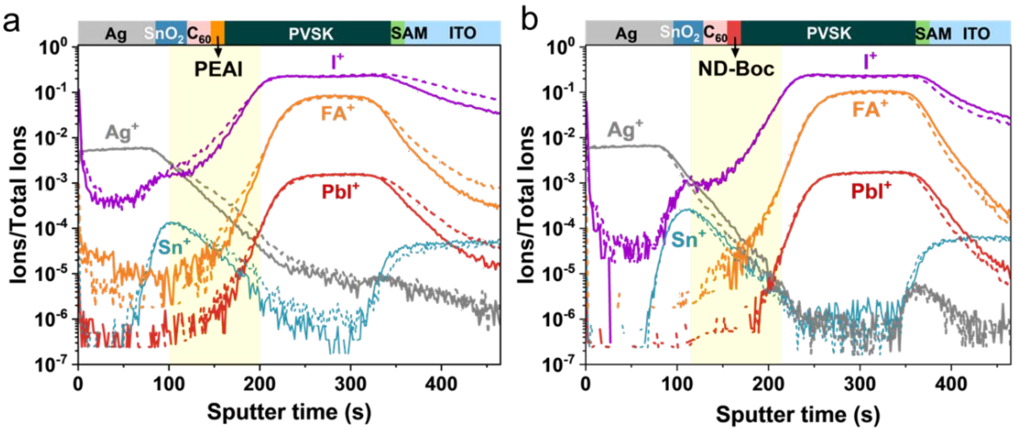

Figure S29. (a) ToF-SIMS of PEAI-modified PSCs before (solid line) and after (dash line) 150 hours of thermal aging at 85°C. (b) ToF-SIMS of ND-Boc modified PSCs before (solid line) and after (dash line) 150 hours of thermal aging at 85°C. Device configuration is ITO/SAM/PVSK/PEAI or ND-Boc/C_{60}/SnO_2/Ag.

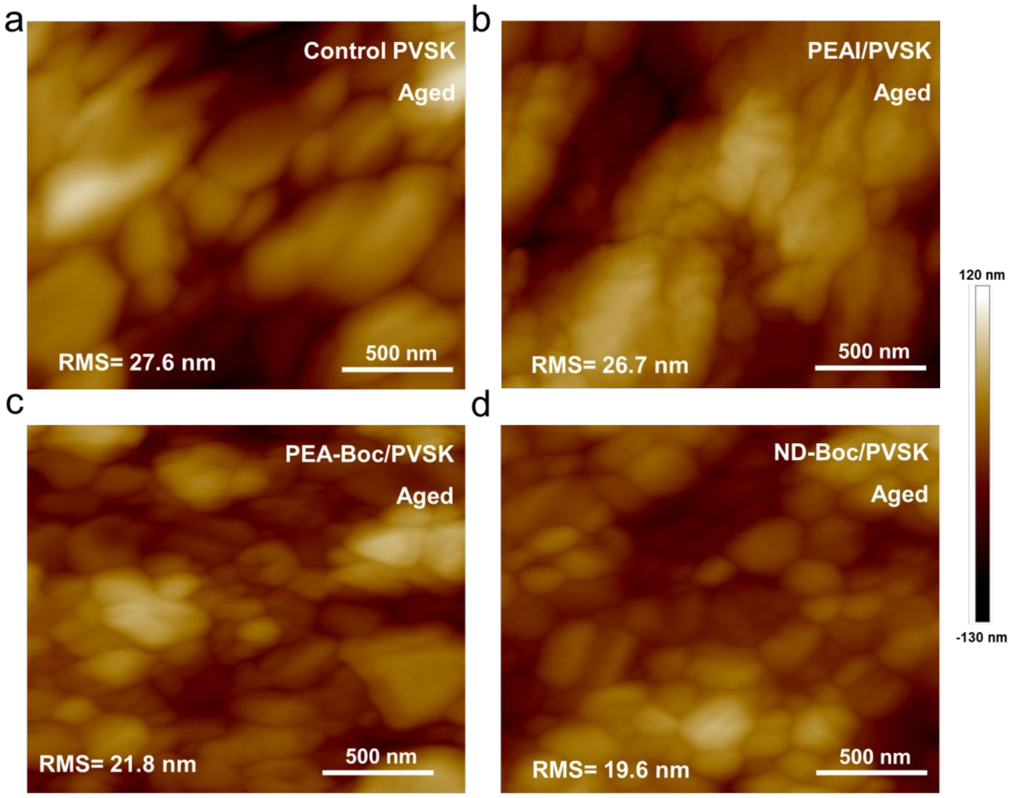

Figure S30. AFM images of films after 90-hour 65°C thermal heating and 1-sun light exposure in N_2 environment. (a) Perovskite films without passivation, (b) PEAI passivated perovskite films, (c) PEA-Boc passivated perovskite films, and (d) ND-Boc passivated perovskite films.

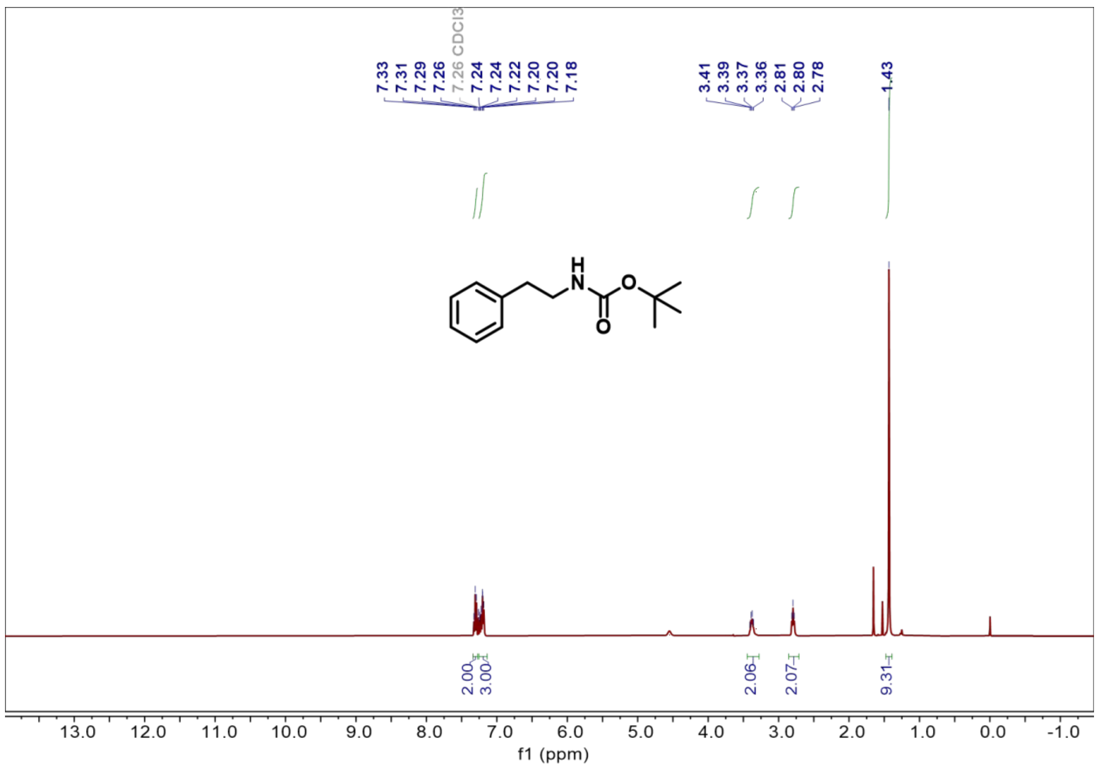

Figure S31. ^1H NMR (400M Hz, $CDCl_3$) of PEA-Boc.

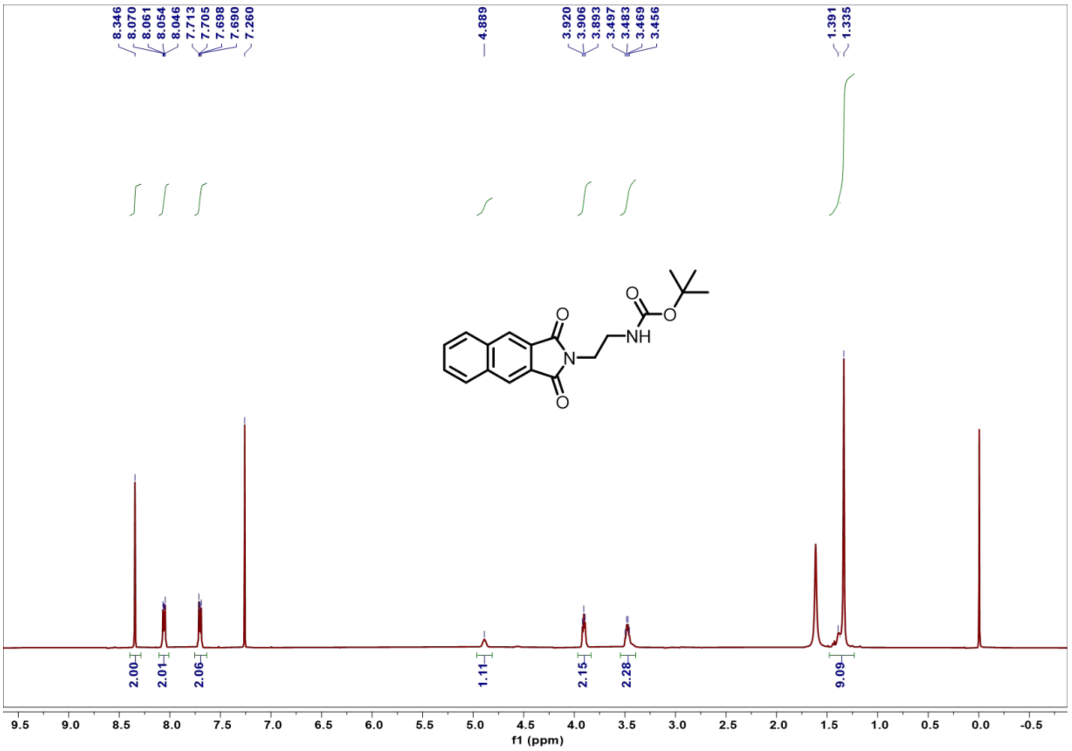

Figure S32. ^{1}H NMR (400M Hz, $CDCl_3$) of ND-Boc.

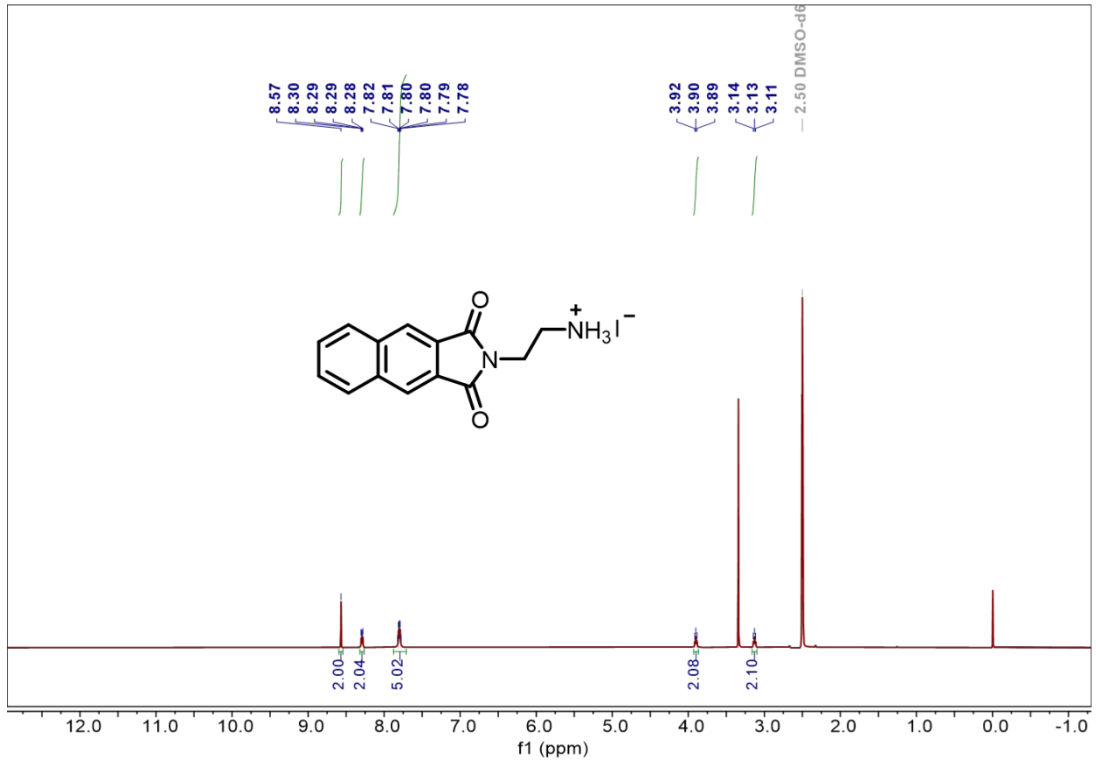

Figure S33. ^{1}H NMR (400M Hz, DMSO-d6) of NDI.

4. Supporting Tables.

Table S1. Crystal data and structure refinement for PEA-Boc.

**Identification code**	103124a_sun	
**Chemical formula**	$C_{13}H_{19}NO_2$	
**Formula weight**	221.29 g/mol	
**Temperature**	213(2) K	
**Wavelength**	1.54178 Å	
**Crystal system**	monoclinic	
**Space group**	P 1 21/c 1	
**Unit cell dimensions**	a = 42.4952(9) Å	α = 90°
	b = 5.26820(10) Å	β = 96.9010(10)°
	c = 11.4895(2) Å	γ = 90°
**Volume**	2553.56(9) $Å^3$	
**Z**	8	
**Density (calculated)**	1.151 g/cm^3	
**Absorption coefficient**	0.615 mm^{-1}	
**F(000)**	960	
**Theta range for data collection**	3.14 to 74.62°	
**Index ranges**	-53<=h<=53, -6<=k<=6, -13<=l<=14	
**Reflections collected**	29840	
**Independent reflections**	5244 [R(int) = 0.1008]	
**Coverage of independent reflections**	99.8%	
**Absorption correction**	Multi-Scan	
**Structure solution technique**	direct methods	
**Structure solution program**	SHELXT 2014/5 (Sheldrick, 2014)	
**Refinement method**	Full-matrix least-squares on F^2	
**Refinement program**	SHELXL-2016/6 (Sheldrick, 2016)	
**Function minimized**	$\Sigma w(F_o^2 - F_c^2)^2$	
**Data / restraints / parameters**	5244 / 0 / 303	
**Goodness-of-fit on F^2**	1.010	
**Δ/σ_{max}**	0.001	
**Final R indices**	4195 data; $I>2\sigma(I)$	R1 = 0.0698, wR2 = 0.1805
	all data	R1 = 0.0814, wR2 = 0.1952
**Weighting scheme**	$w=1/[\sigma^2(F_o^2)+(0.1378P)^2+0.1165P]$	
	where $P=(F_o^2+2F_c^2)/3$	
**Largest diff. peak and hole**	0.370 and -0.371 $eÅ^{-3}$	
**R.M.S. deviation from mean**	0.080 $eÅ^{-3}$	

Table S2. Crystal data and structure refinement for ND-Boc.

**Identification code**	101724a_sun	
**Chemical formula**	$C_{19}H_{20}N_2O_4$	
**Formula weight**	340.37 g/mol	
**Temperature**	213(2) K	
**Wavelength**	1.54178 Å	
**Crystal system**	monoclinic	
**Space group**	P 1 21/n 1	
**Unit cell dimensions**	a = 5.1199(3) Å	α = 90°
	b = 17.4932(9) Å	β = 90.034(3)°
	c = 19.0443(10) Å	γ = 90°
**Volume**	1705.67(16) $Å^3$	
**Z**	4	
**Density (calculated)**	1.325 g/cm^3	
**Absorption coefficient**	0.770 mm^{-1}	
**F(000)**	720	
**Theta range for data collection**	2.32 to 74.55°	
**Index ranges**	-6<=h<=6, -21<=k<=20, -23<=l<=23	
**Reflections collected**	17122	
**Independent reflections**	3484 [R(int) = 0.1167]	
**Coverage of independent reflections**	99.9%	
**Absorption correction**	Multi-Scan	
**Structure solution technique**	direct methods	
**Structure solution program**	SHELXT 2014/5 (Sheldrick, 2014)	
**Refinement method**	Full-matrix least-squares on F^2	
**Refinement program**	SHELXL-2016/6 (Sheldrick, 2016)	
**Function minimized**	Σ w(F_o^2 - F_c^2)^2	
**Data / restraints / parameters**	3484 / 0 / 233	
**Goodness-of-fit on F^2**	1.052	
**Final R indices**	2976 data; I>2σ(I)	R1 = 0.0658, wR2 = 0.1637
	all data	R1 = 0.0725, wR2 = 0.1721
**Weighting scheme**	$w=1/[\sigma^2(F_o^2)+(0.1069P)^2+0.0800P]$ where $P=(F_o^2+2F_c^2)/3$	
**Largest diff. peak and hole**	0.355 and -0.358 $eÅ^{-3}$	
**R.M.S. deviation from mean**	0.083 $eÅ^{-3}$	

Table S3. Space-charge-limited current (SCLC) analysis of devices prepared with different surface treatments. The V_{TFL} was extrapolated from SCLC plots, and trap density was calculated based on V_{TFL}.

	V_{TFL} (V)	Trap density (cm^{-3})
Control	0.42	2.73×10^{15}
PEAI/PVSK	0.35	2.21×10^{15}
PEA-Boc/PVSK	0.45	2.93×10^{15}
ND-Boc/PVSK	0.21	1.37×10^{15}

Table S4. PV device (1.8 eV) parameters.

	V_{OC} (V)	J_{SC} (mA/cm^2)	*FF* (%)	PCE (%)
ND-Boc reverse scan	1.34	17.08	81.76	18.68
ND-Boc forward scan	1.33	17.00	81.16	18.37
Control reverse scan	1.22	16.20	79.74	15.76
Control forward scan	1.21	16.18	78.39	15.30

References

(1) Jiang, W.; Li, F.; Li, M.; Qi, F.; Lin, F. R.; Jen, A. K. -Y. Π-Expanded Carbazoles as Hole-Selective Self-Assembled Monolayers for High-Performance Perovskite Solar Cells. *Angewandte Chemie* 2022, *134* (51), 1–6. https://doi.org/10.1002/ange.202213560.

(2) Neese, F. The ORCA Program System. *Wiley Interdiscip Rev Comput Mol Sci* 2012, *2*, 73–78. https://doi.org/10.1002/wcms.81.

(3) Clark, S. J.; Segall, M. D.; Pickard, C. J.; Hasnip, P. J.; Probert, M. I. J.; Refson, K.; Payne, M. C. First Principles Methods Using CASTEP. *Zeitschrift fur Kristallographie* 2005, *220* (5–6), 567–570. https://doi.org/10.1524/zkri.220.5.567.65075.

(4) Perdew, J. P.; Burke, K.; Ernzerhof, M. Generalized Gradient Approximation Made Simple. *Phys. Rev. Lett.* 1996, *77* (18), 3865–3868. https://doi.org/10.1103/PhysRevLett.77.3865.

(5) GRIMME，STEFAN. Semiempirical GGA-Type Density Functional Constructed with a Long-Range Dispersion Correction. *J Comput Chem* 2012, *27* (15), 1787–1799. https://doi.org/10.1002/jcc.
